# Supplementary material for: Empowering parents to optimize feeding practices with preschool children (EPO-Feeding): A study protocol for a feasibility randomized controlled trial
Source: PLoS One. 2024 Jun 3;19(6):e0304707. doi: 10.1371/journal.pone.0304707 (PMC11146728; doi:10.1371/journal.pone.0304707)
Supplement: S1 File — (PDF) [file pone.0304707.s002.pdf]

Study protocol

Filter Questions

1 Is your study considered research as defined in the guidance icon information?

- (•) Yes
- ( ) No

2 Does your study require any of the following external ethical reviews? NHS REC; Social Care REC or Ministry of Defence REC
See guidance icon for further information on NHS REC, Social Care REC and MOD REC review remit.

- ( ) Yes
- (•) No

Data Collection

3 Select one category from the list below (categories are defined in the guidance icon).

My study involves:

- (•) a) Only primary data collection involving human subjects.
- ( ) b) Use of identifiable human subject data that is not in the public domain. This includes analysis of previously collected data (including human tissue) and/or the use of existing data that has not previously been used for research purposes (such as data taken from private social media groups)
- ( ) c) Both primary data collection involving human subjects and the use of identifiable pre-existing human subject data not in the public domain, as outlined above
- ( ) d) Data collection not involving any of the above but presenting sensitive issues
- ( ) e) None of the above

4 Risk Checklist: Please indicate if your study involves any of the following risks:

- [ ] a) The research involves participants who are vulnerable or unable to give informed consent or in a dependent position.
- [ ] b) Participants will take part in the study without their consent or knowledge at the time of participation or deception of some kind will be involved.
- [ ] c) The research topic may lead participants to disclose their involvement in activities that are illegal, could make them the target of personal or professional reprisals, or otherwise represent a threat to themselves or others.
- [x] d) The study may induce psychological stress or anxiety, or produce humiliation or cause harm or negative consequences beyond the risks encountered in a participant's usual everyday life.
- [ ] e) Participation in this research may identify urgent mental health risks, including, but not limited to, suicidal ideation and/or self-harm intent.
- [ ] f) There is a foreseeable likelihood that a participant's capacity to give fully informed consent may diminish throughout the course of the project i.e. early stage dementia, brain injury etc.
- [ ] g) The study involves imaging techniques such as MRI scans or ultrasound.
- [ ] h) The study involves sources of non-ionising radiation (e.g. lasers)
- [ ] i) The study involve physically invasive procedures or the collection of bodily materials (including collection of human tissue for purposes such as DNA/RNA analysis)
- [ ] None of the above.

5 Does the study involve the recruitment of participants under the age of 16?

- ( ) Yes
- (•) No

Based on your answers to the above filter questions your research has been categorised as High Risk

You can now access an overview of the available sections of the application by selecting the navigate tile in the action panel on the left. Alternatively you can proceed through each section of the application by selecting the next tile.

Upon submission will be subject to review at the next relevant Research Ethics Subcommittee meeting. Meeting dates and submission deadlines can be found here

Section A: General Information

A Applicant Details

First Name	Surname
Jian	Wang

Department: Florence Nightingale Faculty of Nursing, Midwifery & Palliative Care

KCL Email: jian.3.wang@kcl.ac.uk

A2 Applicant Status

MPhil / PhD/ Specialist Doctorate

A5 Is King's College London the lead research sponsor? ◉ Yes ○ No

A7 Faculty/Institute/School

Please refer to the information icon if you are unsure of your Faculty/Institute/School.

Nursing and Midwifery

A8 Course/Qualification

MPhil/PhD

Section B: Project Information

B1 Project Title
A working title that accurately reflect the aims of the project.

Empowering Parents to Optimize Feeding Practices (EPO Feeding): a Feasibility and Acceptability Randomized Controlled Trial

B2 Anticipated start date for the collection of data: 04/12/2023

B3 Expected completion date of the project: 17/03/2024

B4 Is this a funded project?

- ○ Yes
- ◉ No

B5 Please provide a summary of your project aims and objectives written in lay language that will be easily understandable to non-academic readers and non-specialists in your field. This summary should ideally be prefaced by the research question you hope to investigate in relation to your project, hypotheses to be tested, as well as a brief explanation of the academic background to the study.

Please Note: Applications to the Health Faculties RESC should include a full list of references/citations to back up the academic/scientific justification of the project.

Background and Rational

Feeding practices refer to specific practices or strategies that caregivers employ to manage what, when, and how much their children eat and to shape their children's eating (Boots, Tiggemann, & Corsini, 2018; Vaughn, Tabak, Bryant, & Ward, 2013; Ventura & Birch, 2008). There are two types of feeding practices: non-responsive and responsive feeding practices (Jansen, Williams, Mallan, Nicholson, & Daniels, 2018; Savage, Rollins, Kugler, Birch, & Marini, 2017; Shi et al., 2017). Non-responsive feeding practices (also known as coercive control), such as pressure to eat, restriction of food, and use of food as a reward (Vaughn et al., 2016), have been the most studied and raised concerns owing to their close links with children’s obesity (Beckers, Karssen, Vink, Burk, & Larsen, 2021; Boucheron & Bhopal, 2020; Dev, McBride, Fiese, Jones, & Cho, 2013; Faith, Scanlon, Birch, Francis, & Sherry, 2004; Ruzicka, Darling, & Sato, 2020). Positive relationships between non-responsive feeding practices and child weight status have been consistently reported (Beckers et al., 2021; Ruzicka et al., 2020). A recent meta-analysis of 51 studies, with 17431 parent-child dyads reported that the use of controlling feeding practices by caregivers was associated with a greater risk of child obesity (Ruzicka et al., 2020). In contrast, responsive feeding practices have been identified as having a protective effect against childhood overweight and obesity and are associated with reduced risk of overweight and obesity (Adams et al., 2018; Matvienko-Sikar et al., 2018). Unlike adults, children cannot choose the environment in which they live or the food they eat. Caregivers’ feeding practices are therefore critical in shaping children’s eating habits and are a key target in efforts to reduce childhood overweight and obesity (Golan & Crow, 2004; Moore, Tapper, & Murphy, 2010). However, the findings from our systematic review showed that few studies focused on optimizing/changing caregivers’ feeding practices with preschool children and used them as primary outcomes. It may be attributed to their interest in child nutritional-related variables (e.g., obesity prevention and management and child healthy eating promotion) in most articles (Hart et al., 2016; Haines et al., 2016; Leung et al., 2014). Therefore, there is a need to develop an intervention program focused on the most effective ways to optimize feeding practices.

Due to their close link to childhood overweight and obesity, many studies have examined factors related to feeding practices (Chang, Mendelsohn, Fierman, Au, & Messito, 2017; Eichler & Schmidt, 2019; Francis, Hofer, & Birch, 2001; Freitas et al., 2019; Robertson, Dempster, Doherty, & Sharpe, 2022; Ruzicka et al., 2020). Recent studies have reported that caregivers’ perception of child weight (weight perception) is associated with their feeding practices, which has been also confirmed in my previous cross-sectional study (Wang et al., 2022). However, research has shown that caregivers of young children do not perceive overweight or obesity as a health threat (Doolen, Alpert, & Miller, 2009; Rietmeijer-Mentink, Paulis, van Middelkoop, Bindels, & van der Wouden, 2013). Instead, they prefer chubby children and tend to use food as an educational and emotional tool (Jiang et al., 2007). Recent findings reported a high percentage of misperception of child weight, with caregivers tending to underestimate children’s weight, especially in low social economic areas (Lundahl, Kidwell, & Nelson, 2014; Queally et al., 2018; Rietmeijer-Mentink et al., 2013; Wang et al., 2022). In China, where mothers or grandparents are commonly the primary caregivers of children, studies have reported over half of the caregivers underestimated the actual weight of their overweight children (Wang et al., 2022). More problematic, when caregivers cannot recognize their preschool children’s weight problems, they may not implement effective feeding practices to control their children’s weight. In addition, targeting interventions on caregivers’ misperception of their child’s weight has already been confirmed to be effective (Flores-Peña, He, Sosa, Avila-Alpirez, & Trejo-Ortiz, 2018; Gomes, Barros, Pereira, & Roberto, 2018; Zacarías, Shamah-Levy, Elton-Puente, Garbus, & García, 2019). Thus, there is a need to include the component of improving caregivers’ accurate perception of child weight as one of the components of intervention on optimizing their feeding practices.

The aim of the whole project is to develop and test the feasibility of an intervention to improve caregivers' perception of preschool child weight and their feeding practices in China.

In this ethical application, we will apply ethics for a feasibility randomized controlled trial (the final phase of my PhD project). The objectives of this feasibility RCT are to 1) test the feasibility and acceptability of the intervention program (further modify and refine the intervention program); 2) test the potential effects of the intervention program on parental feeding practices, perception of child weight, parenting sense of competence; and child eating behaviors and BMI-Z scores.

List of references

Adams, E. L., Marini, M. E., Stokes, J., Birch, L. L., Paul, I. M., & Savage, J. S. (2018). INSIGHT responsive parenting intervention reduces infant's screen time and television exposure. Int J Behav Nutr Phys Act, 15(1), 24. doi:10.1186/s12966-018-0657-5
Beckers, D., Karssen, L. T., Vink, J. M., Burk, W. J., & Larsen, J. K. (2021). Food parenting practices and children's weight outcomes: A systematic review of prospective studies. Appetite, 158, 105010. doi:10.1016/j.appet.2020.105010
Boots, S. B., Tiggemann, M., & Corsini, N. (2018). "That's enough now!": A prospective study of the effects of maternal control on children's snack intake. Appetite, 126, 1-7. doi:10.1111/apa.14299
Boucheron, P., & Bhopal, S. (2020). Observed feeding behaviours and effects on child weight and length at 12 months of age: Findings from the SPRING cluster-randomized controlled trial in rural India. 15(8), e0237226. doi:10.1371/journal.pone.0237226
Chang, L. Y., Mendelsohn, A. L., Fierman, A. H., Au, L. Y., & Messito, M. J. (2017). Perception of Child Weight and Feeding Styles in Parents of Chinese-American Preschoolers. J Immigr Minor Health, 19(2), 302-308. doi:10.1007/s10903-016-0541-9
Dev, D. A., McBride, B. A., Fiese, B. H., Jones, B. L., & Cho, H. (2013). Risk factors for overweight/obesity in preschool children: an ecological approach. Child Obes, 9(5), 399-408. doi:10.1089/chi.2012.0150
Doolen, J., Alpert, P. T., & Miller, S. K. (2009). Parental disconnect between perceived and actual weight status of children: a metasynthesis of the current research. J Am Acad Nurse Pract, 21(3), 160-166. doi:10.1111/j.1745-7599.2008.00382.x
Eichler, J., & Schmidt, R. (2019). Stability, Continuity, and Bi-Directional Associations of Parental Feeding Practices and Standardized Child Body Mass Index in Children from 2 to 12 Years of Age. 11(8). doi:10.1177/0145445519865170
Faith, M. S., Scanlon, K. S., Birch, L. L., Francis, L. A., & Sherry, B. (2004). Parent-child feeding strategies and their relationships to child eating and weight status. Obes Res, 12(11), 1711-1722. doi:10.1038/oby.2004.212
Flores-Peña, Y., He, M., Sosa, E. T., Avila-Alpirez, H., & Trejo-Ortiz, P. M. (2018). Study protocol: intervention in maternal perception of preschoolers' weight among Mexican and Mexican-American mothers. BMC public health, 18(1), 669. doi:10.1186/s12889-018-5536-0
Francis, L. A., Hofer, S. M., & Birch, L. L. (2001). Predictors of maternal child-feeding style: maternal and child characteristics. Appetite, 37(3), 231-243. doi:10.1006/appe.2001.0427
Freitas, F. R., Moraes, D. E. B., Warkentin, S., Mais, L. A., Ivers, J. F., & Taddei, J. (2019). Maternal restrictive feeding practices for child weight control and associated characteristics. J Pediatr (Rio J), 95(2), 201-208. doi:10.1016/j.jped.2017.12.009
Golan, M., & Crow, S. (2004). Targeting parents exclusively in the treatment of childhood obesity: long-term results. Obes Res, 12(2), 357-361. doi:10.1038/oby.2004.45
Gomes, A. I., Barros, L., Pereira, A. I., & Roberto, M. S. (2018). Effectiveness of a parental school-based intervention to improve young children's eating patterns: a pilot study. Public Health Nutr, 21(13), 2485-2496. doi:10.1017/s1368980018000952
Haines J, Rifas-Shiman SL, Gross D, McDonald J, Kleinman K, Gillman MW. Randomized trial of a prevention intervention that embeds weight-related messages within a general parenting program. Obesity (Silver Spring). 2016;24(1):191-9.
Hart LM, Damiano SR, Paxton SJ. Confident body, confident child: A randomized controlled trial evaluation of a parenting resource for promoting healthy body image and eating patterns in 2- to 6-year old children. Int J Eat Disord. 2016;49(5):458-72.
Jansen, E., Williams, K. E., Mallan, K. M., Nicholson, J. M., & Daniels, L. A. (2018). Bidirectional associations between mothers' feeding practices and child eating behaviours. Int J Behav Nutr Phys Act, 15(1), 3. doi:10.1186/s12966-018-0644-x
Jiang, J., Rosenqvist, U., Wang, H., Greiner, T., Lian, G., & Sarkadi, A. (2007). Influence of grandparents on eating behaviors of young children in Chinese three-generation families. Appetite, 48(3), 377-383. doi:10.1016/j.appet.2006.10.004
Leung C, Tsang S, Heung K. The Effectiveness of Healthy Start Home Visit Program: Cluster Randomized Controlled Trial. Research on Social Work Practice. 2014;25(3):322-33.
Lundahl, A., Kidwell, K. M., & Nelson, T. D. (2014). Parental underestimates of child weight: a meta-analysis. Pediatrics, 133(3), e689-703. doi:10.1542/peds.2013-2690
Matvienko-Sikar, K., Toomey, E., Delaney, L., Harrington, J., Byrne, M., & Kearney, P. M. (2018). Effects of healthcare professional delivered early feeding interventions on feeding practices and dietary intake: A systematic review. Appetite, 123, 56-71. doi:10.1016/j.appet.2017.12.001
Moore, S. N., Tapper, K., & Murphy, S. (2010). Feeding goals sought by mothers of 3-5-year-old children. Br J Health Psychol, 15(Pt 1), 185-196. doi:10.1348/135910709x447668
Queally, M., Doherty, E., Matvienko-Sikar, K., Toomey, E., Cullinan, J., Harrington, J. M., & Kearney, P. M. (2018). Do mothers accurately identify their child's overweight/obesity status during early childhood? Evidence from a nationally representative cohort study. Int J Behav Nutr Phys Act, 15(1), 56. doi:10.1186/s12966-018-0688-y
Rietmeijer-Mentink, M., Paulis, W. D., van Middelkoop, M., Bindels, P. J., & van der Wouden, J. C. (2013). Difference between parental perception and actual weight status of children: a systematic review. Matern Child Nutr, 9(1), 3-22. doi:10.1111/j.1740-8709.2012.00462.x
Robertson, M. D. A., Dempster, S., Doherty, L., & Sharpe, H. (2022). Exploring the association between parental anti-fat attitudes and restrictive feeding practices in a British and Irish sample. Appetite, 168, 105755. doi:10.1016/j.appet.2021.105755
Ruzicka, E. B., Darling, K. E., & Sato, A. F. (2020). Controlling child feeding practices and child weight: A systematic review and meta-analysis. Obes Rev. doi:10.1111/obr.13135
Savage, J. S., Rollins, B. Y., Kugler, K. C., Birch, L. L., & Marini, M. E. (2017). Development of a theory-based questionnaire to assess structure and control in parent feeding (SCPF). Int J Behav Nutr Phys Act, 14(1), 9. doi:10.1186/s12966-017-0466-2
Shi, C., Li, N., Dong, J., Wang, L., Li, X., Ji, C., . . . Zhang, M. (2017). Association between maternal nonresponsive feeding practice and child's eating behavior and weight status: children aged 1 to 6 years. Eur J Pediatr, 176(12), 1603-1612. doi:10.1007/s00431-017-3007-8
Vaughn, A. E., Tabak, R. G., Bryant, M. J., & Ward, D. S. (2013). Measuring parent food practices: a systematic review of existing measures and examination of instruments. Int J Behav Nutr Phys Act, 10, 61. doi:10.1186/1479-5868-10-61
Vaughn, A. E., Ward, D. S., Fisher, J. O., Faith, M. S., Hughes, S. O., Kremers, S. P., . . . Power, T. G. (2016). Fundamental constructs in food parenting practices: a content map to guide future research. Nutr Rev, 74(2), 98-117. doi:10.1093/nutrit/nuv061
Ventura, A. K., & Birch, L. L. (2008). Does parenting affect children's eating and weight status? Int J Behav Nutr Phys Act, 5, 15. doi:10.1186/1479-5868-5-15
Wang, J., Zhu, D., Cheng, X., Liuzhou, Y., Zhu, B., Montgomery, S., & Cao, Y. (2022). Maternal perception of child weight and concern about child overweight mediates the relationship between child weight and feeding practices. Public Health Nutr, 1-10. doi:10.1017/s1368980022000040
Zacarías, G., Shamah-Levy, T., Elton-Puente, E., Garbus, P., & García, O. P. (2019). Development of an intervention program to

prevent childhood obesity targeted to Mexican mothers of school-aged children using intervention mapping and social cognitive theory. Eval Program Plann, 74, 27-37. doi:10.1016/j.evalprogplan.2019.02.008

B6 Where will the research be conducted? i.e in a facility within the college, in a private organisation, in a public place etc

The study will be conducted at two kindergartens in Yangzhou, Jiangsu, China.

B7 If outside of the UK, please state the country/countries in which data collection is expected to occur.

China

B8 Selection of methodology from list: (select each that applies)

- ☑ Questionnaires
- ☑ Semi-structured interviews
- ☐ Unstructured Interviews
- ☐ Focus Groups
- ☑ Observation
- ☐ Clinical Procedures or Interventions
- ☑ Non-clinical Procedures or Interventions
- ☑ Randomised Controlled Trial
- ☐ Oral history
- ☐ Analysis of pre-existing data from human participants
- ☑ Audio/video recording or photography in a public place
- ☑ Audio/video recording or photography in a private place
- ☐ Administration of substances (including food)
- ☐ Behavioural/Cognitive Testing
- ☐ Other

Please note: If you intend to audio/ video record participants, these recordings are considered identifiable personal data under UK GDPR and therefore must be highlighted in Section E, as well as the relevant recruitment documents.

If you are using any standardised methods for any of the above selected methodologies, please provide an overview of any standardised documentation to be used. Please provide full names and references where appropriate.

1. Process evaluation
Research on using qualitative methods for feasibility and acceptability RCT will be likely of particular importance in refining understanding of how the intervention works and facilitating ongoing adaptation of intervention and evaluation design in preparation for a full trial. Thus, this study will nest a process evaluation, including semi-structured interviews and observation of modules, which aim to extend an in-depth understanding EPO Feeding intervention program for this feasibility and acceptability trial.
1) Semi-structured interviews will be conducted for participants and healthcare professionals who delivered the intervention modules. A topic guide (Appendix 1: interview topic guide) was developed by the research team to address the research questions.
2) The researcher (JW) will observe each module, which aims to expand the immediate response and feedback for each module

and to assess the level of intervention fidelity. The researcher (JW) will also make fieldnotes about the module delivery and response to the modules provided on the program, barriers and enables of the program completion, participants' task engagement, and its overall structure, delivery, and content of the program (Appendix 2: Observation checklist).
3) All modules will be video/audio recorded by a facilitator and checked by two researchers (XXW and JW) to rate against the fidelity checklists and ensure modules are delivered as intended (Appendix 3: Fidelity checklist).

2. Quantitative data/outcomes
-The primary objectives (i.e., the feasibility and acceptability of the EPO Feeding program) will be collected. These included data on recruitment and retention, adherence, and the feasibility of the outcomes which are described below:
Recruitment and retention
• Number of eligible participants approached and consenting to take part, which is randomized, as well as the number of ineligible participants.
• Number of participants who successfully complete modules
• Number of participants lost due to follow-up and dropout rate.
Attendance/Adherence
• Number of (each) modules attended.
• Number of homework/assignments finished.
Acceptability
• An anonymous survey containing eight closed questions (e.g., How would you rate the quality of the program? How valuable was the program in helping you optimize feeding practices?) and one open question (i.e., comments on the experiences or feelings about EPO Feeding program) after the intervention. The survey for acceptability is uploaded (Appendix 7 English version)

-The secondary objective is to test the potential effects of EPO Feeding program. These data will be collected at the baseline and follow-ups. The questionnaires for baseline and follow-up are uploaded (Appendix 4 and 5).
• Demographic and socioeconomic data including children's age, sex; caregivers' role, age, weight, height, education level and household annual income, and number of children will be collected at baseline only.
• Parental feeding practices The Chinese Preschoolers' Caregivers' Feeding Behavior Scale (CPCFBS) will be used to evaluate parental feeding practices [1]. The CPCFBS assesses two types of non-responsive feeding practices: four items of content-restricted feeding (strict limitations on the children's access to foods or opportunities to consume unhealthy foods [2]), three items of pressure to eat (insists, demands, or physical struggles with the child in order to get the child to eat more food [2,3,4]), and three types of responsive feeding practices: four items of monitoring (the extent to oversee their child eating [3]), six items of encouraging healthy eating (the behaviors of encouraging their children to eat more healthy food [1]) and seven items of modelling (modelling of healthy food choice to encourage children to adopt similar behaviors [1]). Two items of use of food as a reward (use of desired food as a method to regulate child eating or behaviors) will be assessed using the Chinese vision of the Child Feeding Questionnaire (C-CFQ) [5]. Each item of CPCFBS and C-CFQ is rated on a 5-point Likert scale. The response options for each item are "always," "usually," "sometimes", "rarely", and "never". Each subscale is calculated by averaging the scores of all the items in that subscale.
• Parental perception of preschool child weight (i.e., self-reported and visual perception) The Chinese version of the Child Feeding Questionnaire (C-CFQ) [5] will be used to measure the self-reported perception of child weight by asking to describe their child's weight status from 5 response options: "underweight", "a little underweight", "about the right weight", "a little overweight", or "overweight" (by words). Parental visual perception of child weight will be assessed by the Parents' Perception of Healthy Weight (PPHW) in the age range 2 to 6 years old [6]. The use of PPHW has been supported conceptually [7] and empirically by studies [8, 9] on the Asian population. The parent will be asked to select one gender an appropriate image/sketch that most closely matches the child's body type on a 7-point scale (1 = a severely underweight child, 4 = an average weight child, 7 = an obese child).
• Parenting Sense of Competence The Chinese version of the Parenting Sense of Competence Scale will be used to assess parental perception of their abilities to manage the demands of parenting [10, 11]. It includes two subscales: 8 items in the Efficacy measuring parental perception of competence in the parenting role and 9 items in the Satisfaction subscale assessing parental satisfaction and comfort with the parenting role [10]. Each item is rated on a 6-point Likert scale, from "Absolutely disagree" to "Absolutely agree". Each subscale is calculated by averaging the scores of all the items in that subscale.
• Child eating behaviors Five common types of child eating behaviors will be assessed by the Chinese Preschoolers' Eating Behavior Questionnaire (CPEBQ) [12], which includes five items of food fussiness (reluctance to try new food or eating limited food [12, 13]), six items of food responsiveness (the desire to eat food when children see or smell food, or were supplied with food [12, 13]), five items of satiety responsiveness (the limited amount of food the child eats in a meal [12, 13]), four items of unhealthy eating habits (the behaviors of child chewing, swallowing, spitting out or throwing, etc. [12]) and five items of initiative eating (the ability of child independent eating [12]). Each item was rated on a 5-point Likert scale. The response options for each item are "always," "usually," "sometimes", "rarely", and "never". Each subscale is calculated by averaging the scores of all the items in that subscale.
• Child weight status According to the World Health Organization (WHO) guidelines, child age-standardized BMI Z-scores were calculated using the software WHO Anthro (for 2- to 5-year-old children) and WHO AnthroPlus (for 5- to 6-year-old children). BMI Z-scores are categorized into three groups: underweight (Z-score < -2), normal weight (-2 ≤ Z-score ≤ 1), overweight (1 < Z-score ≤ 2), and obese (Z-score ＞ 2) [14].

[1] Yuan, J., et al., Development and Preliminary Evaluation of Chinese Preschoolers' Caregivers' Feeding Behavior Scale. J Acad Nutr Diet, 2019. 119(11): p. 1890-1902.
[2] Vaughn, A.E., et al., Fundamental constructs in food parenting practices: a content map to guide future research. Nutr Rev, 2016. 74(2): p. 98-117.
[3] Birch, L.L., et al., Confirmatory factor analysis of the Child Feeding Questionnaire: a measure of parental attitudes, beliefs and practices about child feeding and obesity proneness. Appetite, 2001. 36(3): p. 201-10.
[4] Arlinghaus, K.R., et al., Exploratory factor analysis of The Comprehensive Feeding Practices Questionnaire (CFPQ) in a low-income hispanic sample of preschool aged children. Appetite, 2019. 140: p. 82-90.
[5] Zheng L, Song D, Chen C, Li F, Zhu D. Reliability and validity of a Chinese version of Child Feeding Questionnaire among parents of preschoolers. Chinese journal of child health care. 2016;24(10):1019-23

[6] Collins, M.E., Body figure perceptions and preferences among preadolescent children. International Journal of Eating Disorders, 1991. 10(2): p. 199-208.
[7] Mareno, N., Parental perception of child weight: a concept analysis. J Adv Nurs, 2014. 70(1): p. 34-45.
[8] Pallan, M.J., et al., Body image, body dissatisfaction and weight status in South Asian children: a cross-sectional study. BMC Public Health, 2011. 11: p. 21.
[9] Wang, G.H., T.X. Tan, and C.S. Cheah, Preschool-Age Chinese Children's Weight Status: WHO Classification, Parent Ratings, Child/Family Characteristics. J Pediatr Nurs, 2017. 33: p. 63-69.
[10] Ngai, F.W., S. Wai-Chi Chan, and E. Holroyd, Translation and validation of a chinese version of the parenting sense of competence scale in chinese mothers. Nurs Res, 2007. 56(5): p. 348-54.
[11] Johnston, C. and E.J. Mash, A Measure of Parenting Satisfaction and Efficacy. Journal of Clinical Child Psychology, 1989. 18(2): p. 167-175.
[12] Jiang, X., et al., Development and preliminary validation of Chinese preschoolers' eating behavior questionnaire. PLoS One, 2014. 9(2): p. e88255.
[13] Wardle, J., et al., Development of the Children's Eating Behaviour Questionnaire. J Child Psychol Psychiatry, 2001. 42(7): p. 963-70
[14] de Onis, M., et al., [WHO growth standards for infants and young children]. Arch Pediatr, 2009. 16(1): p. 47-53.

B9 Provide an explanation in lay language outlining each methodology of the study, as identified in question B8.

The development process of EPO Feeding program is according to MRC framework [49], including systematic reviews, qualitative interviews, cross-sectional study, and feasibility and acceptability RCT. In this study, we will conduct the feasibility and acceptability RCT. The development process for this feasibility trial is uploaded in Appendix Figure 1.

Recruitment and consent process:
The recruitment and consent process for participants is the same as outlined in section C4 of this form. (Appendix figure 2)
Participant Eligibility Criteria:
The inclusion and exclusion criteria for participants are the same as outlined in sections C2a and C2b of this form.

Study design
EPO Feeding program involves several components, targeting optimizing parental feeding practices in China. A two-arm feasibility and acceptability RCT with three repeated measures will be used to test the feasibility and acceptability of EPO Feeding program. After baseline assessment, parents who are responsible for their child's eating behaviors and family food environment will be randomly assigned to one of the two conditions: one experimental group (EPO Feeding program and standard care) and one control group (standard care). The four weekly modules of the multicomponent intervention curriculum include four modules. After finishing the program, parents complete the measures immediately and one-month follow-up. Semi-structured interviews will be conducted with parents to support the process evaluation of the 4-week intervention program after the intervention.
Randomization
The randomization will be performed by an independent researcher (XXW) not connected with either participant recruitment or data collection. After completing the baseline assessment, parents will be randomized and allocated to one of the conditions through a concealed computerized random number generator by randomization.com, with an equal allocation ratio (1:1). Selection bias will be minimized by ensuring all participants eligible and recruited have equal opportunity of being allocated to each study group and follow-up completed. Due to the nature of the study, only the research members who collect and analyze the data from participants can be masked to the randomization. At the follow-up data collection time points, child height and weight measurements, and follow-up questionnaires will be taken and distributed by a trained kindergarten healthcare staff blinded to group allocation. Unmasking will not occur until databases are closed, and the main analysis has been completed.
Intervention group
Participants in the intervention group will receive EPO Feeding program and standard care. The EPO Feeding program is a 4-module intervention program focused on optimizing parental feeding practices. The components and contents of the EPO Feeding program are presented in Appendix 6. Two healthcare professionals in the Department of Child Health in the local maternal and child health hospital will be trained to deliver the EPO Feeding program. Before each module, they will pre-present the module to the researcher (JW) to ensure their presentation is in accordance with the EPO Feeding intervention manual via VooV Meeting, which will be recorded and sent to parents in the control group after all follow-ups. Considering the effectiveness and cost-effectiveness of the intervention [1], we will divide participants in the intervention group into two groups (15-18 parents per group) to attend the modules separately. Each weekly module will last 45-60 min in the kindergarten classroom, incorporating lesson/session (i.e., slides shows and handouts), group discussion, uptake of key messages, supplementary materials (e.g., stories, key messages and learning videos) to improve the acquisition of participants' knowledge, skills, and behaviors. Each module will take place twice every week (Friday: 12.30-13.30 PM; Saturday: 09.00-10.00 AM) at the kindergarten premises after receiving approval from the gatekeeper. Parents in the intervention group can choose one regular time to attend the module every week. After each module, homework activities will be assigned to participants to help reinforce their knowledge, skills, and behaviors. Participants will receive weekly messages via WeChat reminding them to attend the module with brief information. On the other hand, a WeChat group will be built to facilitate parental involvement, learning, and communication. The WeChat group will be monitored by two healthcare professionals in the areas of child health and nutrition. You can share your feelings, experiences, ideas or practices relevant to each module in the WeChat group. It is

completely voluntary but encouraged because other parents may find them helpful for their positive behavior change and two HCPs can provide feedback. All shared information in the WeChat group will not be used for any report/publication. Except for baseline assessment (T0), parents in the intervention group will receive text messages about their preschool child's actual weight status a week before the assessment of each time point from a kindergarten healthcare staff who is not involved in collecting child weight and height and is not blind to group allocation. Participants will also be informed that they could make contact via WeChat/phone call if they have related questions or concerns at any time. In addition, participants continue to receive infographics summarising the key points from each of the modules every week via WeChat group at the end of the program until the one-month follow-up.

Control group
Parents involved in the control group will receive standard care, including the printed materials of child health-related dietary recommendations published by Chinese government/Nutrition Society. These materials will also be distributed to the participants in the intervention group. After the final data collection at one-month follow-up, participants from this group will be offered the complete material package of the EPO Feeding program, a book (related to child eating) as a gift and their child's weight status measured at the final time point and provided access to pre-recorded modules by healthcare professionals as an incentive, but they will not be included in the WeChat group.

Data collection:
The schedule of enrolment, interventions, and assessments is shown in Appendix Figure 3.
- Quantitative data
Demographic and socioeconomic data including children's age, sex; caregivers' role, age, weight, height, education level and household annual income, and number of children will be collected at baseline only. A survey related to the trial acceptability will be assessed immediately after the intervention (Appendix 7). Quantitative data including parental feeding practices, perception of child weight, parenting sense of competence; and child eating behaviors will be collected from participants at three-time points: baseline (T0), immediately after the intervention (T1), and one month after the intervention (T2). The kindergarten healthcare staff who is unaware of participants' group allocation will be trained to distribute and collect the questionnaires and measure children's weight and height at each time point in the kindergartens. The questionnaire will take approximately 15 minutes.

The questionnaire also includes some identified information: preschool child student ID number, participant contact details (phone number). We need to assess child's actual weight and height at the kindergarten at each time point after getting the approval of kindergarten gatekeeper. Except for baseline assessment (T0), parents in the intervention group will receive text messages about their preschool child's actual weight status a week before the assessment of each time point from a kindergarten healthcare staff who is not involved in collecting child weight and height and is not blind to group allocation. We will also use child's actual weight status to compare with parental self-reported and visual perception of child's weight separately and then, we can calculate the prevalence of parental misperception of child's weight in this sample.

The included scales have been all used in the Chinese sample and showed moderate to good internal consistency reliability in existing studies including my previous study [2]. The questionnaire is attached in the additional file (Appendix 4 and 5).

- Qualitative data
After completion of modules, the participants will be sent an invitation for semi-structured interview via WeChat messages. If some participants drop out, the researcher (JW) will first send messages to them via WeChat and then ask if they could be interviewed online. If they agree, the semi-structured interview will be conducted after receiving their written informed consent. An interview indicative topic guide (Appendix 1) is informed by the objectives of feasibility and acceptability which will guide the qualitative data collection. Interviews will take place on the kindergarten premises or via phone/WeChat call and will last about 40 min. Two healthcare professionals in the local maternal and child health hospital where we will register our study will deliver the intervention program but will be included in the process evaluation (semi-structured interviews).

Data analysis
- Quantitative data analysis
The primary objectives of this feasibility and acceptability RCT will be addressed via descriptive estimates (e.g., means, standard deviations, and percentages). Measurements of study feasibility include recruitment rate, rates of intervention compliance, and participant retention rates. The total number of participants included in each attendance and assessment will be reported to account for missing data. The retention rate will be calculated as the rate of completion of one-month follow-up. The feasibility of collecting quantitative outcome data includes < 20% missing data at the group level at each time point. The acceptable proportion of enrolled participants who complete all modules will be 80% or greater, given that there's 10%-16% dropout rate of participants in some related RCTs [3,4, 5]. Participants will also rate five elements of the intervention on a 10-point Likert scale will be used to test the acceptability of the intervention. Participant acceptability of the intervention will be considered by a score of six or more, unacceptability will be regarded when participants scored five or less on the Likert scale.

Quantitative secondary objectives will be addressed using exploratory statistical analysis. Intention-to-treat (ITT) principles will be used for parametric data, with all participants analyzed in the group which they are randomized regardless of whether they attend all data collection time-points or complete the intervention. Multiple imputations by chained equations (MICE) will be adopted to impute missing values if cases with < 10% missing data at each data collection time-point because this procedure can use all available variables with complete information in the study [6]. Descriptive statistics were used to describe the participants' characteristics. The characteristics and all dimensions of outcomes (i.e., parental feeding practices, sense of competence, perception of child weight, and child eating behaviors) will be compared between the intervention group and control group using t-tests (for continuous variables) and chi-squared tests (for categories variables). In addition to the ITT analysis, the completers analysis will be conducted using linear mixed models, which include intervention participants who have completed at least 3 modules (>80% of intervention content) and comparison participants who attend all data collection time-points. The linear mixed model regression procedure will be used to test intervention effects on outcomes with continuous variables. The models will include the outcome measures as the dependent

variables, treatment condition (intervention versus control) as the fixed factor, and confounding factors (e.g., parental education level, children's sex, and age) as covariates. McNemar's Test will be used to test the intervention effect on change in the proportion of parental accurate perception of their preschooler's weight status. Statistical significance is set at $P < 0.05$ (two-sided). SPSS Statistics 27.0 (IBM Crop, Armonk, NY, USA) will be used for data coding, cleaning, and analysis.

- Qualitative data analysis

An inductive content analysis approach to thematic analysis using the Thematic Framework Analysis method will be applied to analyze the data [7, 8]. This approach offers researchers a systematic structure to manage, analyze and identify themes consisting of five interconnected stages. The five stages involve familiarization, constructing/identifying a thematic framework, indexing, and sorting, data summary and display and mapping and interpretation [82]. JW will transcribe the recordings. The interview transcripts will be uploaded into NVivo 14. To ensure consistency, the two researchers (JW and XXW) initially indexed 20% of transcripts. The analysis will be conducted in the original Chinese language, and the final themes with examples will be translated into the English language by two researchers (JW and XXW) which could ensure the accuracy and transparency of the data.

[1] Ryder, H.F., et al., Decision Analysis and Cost-effectiveness Analysis. Semin Spine Surg, 2009. 21(4): p. 216-222.
[2] Wang J, Zhu D, Cheng X, et al. Maternal perception of child weight and concern about child overweight mediates the relationship between child weight and feeding practices. Public Health Nutr. 2022:1-10. doi: 10.1017/S1368980022000040.
[3] Hart, L.M., S.R. Damiano, and S.J. Paxton, Confident body, confident child: A randomized controlled trial evaluation of a parenting resource for promoting healthy body image and eating patterns in 2- to 6-year old children. Int J Eat Disord, 2016. 49(5): p. 458-72.
[4] Leung, C., S. Tsang, and K. Heung, The Effectiveness of Healthy Start Home Visit Program: Cluster Randomized Controlled Trial. Research on Social Work Practice, 2014. 25(3): p. 322-333.
[5] Sobko, T., G.T.L. Brown, and W.H.G. Cheng, Does connectedness to nature improve the eating behaviours of pre-schoolers? Emerging evidence from the Play&Grow randomised controlled trial in Hong Kong. Appetite, 2020. 154: p. 104781.
[6] Harel, O. and X.-H. Zhou, Multiple imputation: review of theory, implementation and software. Statistics in Medicine, 2007. 26(16): p. 3057-3077.
[7] Gale, N.K., et al., Using the framework method for the analysis of qualitative data in multi-disciplinary health research. BMC Med Res Methodol, 2013. 13: p. 117.
[8] Srivastava, A. and S. Thomson, Framework Analysis: A Qualitative Methodology for Applied Policy Research. JOAAG, 2008. 4.

If the summary of your methodology would be supported by a flowchart please attach this here (an editable flowchart can be found via the link in the guidance icon)

B10 I confirm that the researcher who will be administering all tests and/or procedures is competent in the methods.

- (●) Yes
- ( ) No

B11 If applicable, please explain any context specific factors relating to your research that may be helpful to the committee when reviewing your application.

For example, an explanation of a novel research method in lay language or an overview of any cultural values or belief systems that have informed your process of obtaining informed consent.

This study has received ethical approval from a local maternal and child health hospital and will be registered in this hospital after receiving ethical approval from King's as the previous phases in my PhD project (i.e., qualitative interviews and cross-sectional study). In this case, two HCPs in the Department of Child Health who are our co-researchers and have professional knowledge and knowledge in this research topic will deliver this intervention program.

Section C: Participants

C1 Detail your projected number of participants and provide justification for this sample size.
Please note: For projects involving mixed methods and/or multiple participant groups, you should provide an estimate of the number of participants taking part in each method.

(1) Parents in this feasibility trial (including parents in the intervention and control group)
As this is a feasibility study, a formal sample size calculation for between-group comparisons of a primary outcome is not appropriate. A sample size of between 25 and 50 is considered reasonable in a feasibility study, to be able to establish feasibility, estimate the difference in retention rates with accuracy, and attain an appropriate standardized effect size (0.15-0.3) [1, 2]. The target recruitment for this feasibility RCT will be 70 (i.e., 35 participants per group), allowing for a 20% loss to follow-up rate and for at least 56 participants to complete the study.

(2) Parents in the process evaluation (semi-structured interviews)
Regarding the semi-structured interviews for process evaluation, a purposive sampling strategy will be used to recruit a variety of participants with different characteristics and backgrounds. 10-12 parents and two healthcare professionals will be recruited.

[1] Whitehead, A.L., et al., Estimating the sample size for a pilot randomized trial to minimize the overall trial sample size for the external pilot and main trial for a continuous outcome variable. Stat Methods Med Res, 2016. 25(3): p. 1057-73.
[2] Teresi, J.A., et al., Guidelines for Designing and Evaluating Feasibility Pilot Studies. Med Care, 2022. 60(1): p. 95-103.

(3) Healthcare professionals in the process evaluation (semi-structured interviews)
These two HCPs (co-researchers) are in the child health department of the local maternal and child health hospital where we will register our study and will deliver the intervention program. After the intervention, the researcher (JW) will ask them to participate in the process evaluation to understand their views and suggestions for this intervention program (semi-structured interviews).

C2a What are the Inclusion Criteria? Where appropriate explain how you will screen your participants. *(The selection criteria should be clearly defined for multiple participant groups)*

-Parents
1) Parents who are the caregivers (i.e., parents who are responsible for the family food environment and their preschool children's eating)
2) One of their children aged 2 to 6 years (if (more than) two preschool children, the parent is instructed to focus on the child whose eating, nutrition or weight status they are more concerned about)
3) Aged ≥18 years
4) Able to provide informed consent
5) Able to speak and write Chinese
-HCPs
1) Able to provide informed consent
2) Aged ≥18 years
3) In the related research/working areas (e.g., nutrition, child health and paediatrics)
4) Able to speak and write Chinese
Note: these two HCPs are part of the research team (co-researchers) already

C2b What are the Exclusion Criteria? Where appropriate explain how you will screen your participants. *(The selection criteria should be clearly defined for multiple participant groups)*

Parents
1) parents with diagnosed severe mental illness such as schizophrenia, uncontrolled bipolar disorder, or mental retardation that would prevent participation in the feasibility intervention.
2) Parents who have eating disorders or are pregnant during the study period.
3) Parents/Their preschool children who participate in another intervention related to child growth and nutrition
4) their preschool children with diseases that influence their eating and nutrition (e.g., diagnosed eating disorders).
5) Parents who participated in our previous semi-structured interviews/focus groups for intervention development.
-No exclusion criteria for HCPs

C2c In line with the inclusion/exclusion criteria outlined above, will you be actively screening out potential participants once they have expressed an interest in taking part in the study?

- (selected) Yes, potential participants will be screened before taking part
- No, potential participants will self-screen in line with the inclusion/exclusion criteria

C2c i) Please outline the process for screening potential participants, including how they will be informed if it is determined that they are not eligible to take part.

Parents who are interested in this study can contact the principal researcher (JW) via work email/ study-specific WeChat/ study-specific phone call (provided on the poster, take-home letters and PIS) for further information. Potential participants contacting JW will be further screened for eligibility. JW will ask parents if they understand the full list of inclusion and exclusion criteria shown on PIS. If they have confusion/questions, JW will provide the explanation until receiving their confirmation for participation or non-participation. JW will avoid sensitive topics and use appropriate words when screening the participants. If eligible, they will be provided with a consent form for their review via email/WeChat. If not eligible, they will be notified during the screening with ineligibility reasons explained to them sensitively. They will be signposted to information and online resources available about child nutrition and eating if they need it. A copy of the ineligibility statement will also be provided for parents at the parent meeting. JW will also let them know if they agree to attend, they should first complete the hard copy of the consent form and baseline questionnaire which can be collected from a healthcare staff within a regular date (around two working days) in the kindergarten’s waiting areas (4.30-5.30 PM off-school period). We will put a box in the kindergarten’s waiting areas for five working days (8:00 AM-5:00 PM every day) after distribution so that they can return the consent form and questionnaires. This healthcare staff will check the box every day and collect the returned consent form and questionnaires. Potential participants will have over 24 hours to decide whether to participate.

C3 What are the upper and lower age limits? Provide justification for these where appropriate.

The study will recruit participants with lower age limits of 18 years and no upper limits applied. In China, 18 years and above are regarded as adults and able to provide informed consent.

C4 How will potential participants be identified and approached?
Please note: If different recruitment methods will be used for participant groups, each group should be separately addressed.

The participants in this feasibility trial are parents of preschool children.
Parents of preschool children will be recruited from two kindergartens in Yangzhou, China after getting the approval of each gatekeeper (head of kindergarten). Participants will be recruited via posters and take-home letters, providing details about the study. Information about the program and how to participate in the study will be also shared through common social networks (i.e., parent meetings). Specifically, after getting the approval, posters inviting caregivers and the contact details of the principal investigator (JW) will be displayed on the main notice boards, and waiting areas at the kindergartens. The healthcare staff who are not the kindergarten teachers in each class will make an announcement for this study and distribute the take-home letters, participant information sheet (PIS) and ineligibility statement to parents in the parent meeting.

This project has received ethical approval from the local maternal and child health hospital in Yangzhou, China, and will be registered after obtaining ethical approval from King's. This hospital takes the responsibility of providing guidance and checking the work related to child health at the local kindergartens. The staff at the hospital have regular contact with the head of each kindergarten and kindergarten healthcare staff. After registration, they will communicate officially with the head of the kindergarten (gatekeeper) and kindergarten healthcare staff about this project. JW will book an appointment for a face-to-face discussion of the research procedures, aims, and potential benefits with each head of the kindergarten. After receiving permission from each gatekeeper, JW will ask some kindergarten healthcare/nursery staff to voluntarily distribute the take-home letters, participant information sheet (PIS) and ineligibility statement at the most recent parent-teacher conference. We will let kindergarten healthcare staff know that it is not mandatory to distribute materials and will not influence their work if they are unavailable. The distribution will happen at the end of the parent-teacher conference and will last 5-10 minutes. In this case, it would not influence the agenda of the conference. We will also obtain permission for this distribution from each gatekeeper at two kindergartens. It is noted that we will not ask the kindergarten teacher in each class to distribute the take-home letters and PIS as they usually have regular contact with parents, which can help mitigate perceived pressure to participate in the context of this recruitment method. During the distribution, these staff will make an announcement that the participation is totally voluntary, and their non-participation will not disadvantage them in any way or influence their child's education. These staff will also notify parents that the take-home letter/poster/PIS contain the contact information that is used for recruitment and communication.

Parents who are interested in this study can contact the principal researcher (JW) via work email/ study-specific WeChat/ study-specific phone call (provided on the poster, PIS and take-home letters) for further information. Potential participants contacting JW will be further screened for eligibility. JW will ask parents if they understand the full list of inclusion and exclusion criteria shown on PIS. If they have confusion/questions, JW will provide further explanation until receiving their confirmation for participation or non-participation. If eligible, they will be provided with the information sheet and consent form for their review via email/WeChat. JW will also let them know if they agree to attend, they should first complete the hard copy of the consent form and baseline questionnaire which can be collected from a healthcare staff within a regular date (around two working days) in the kindergarten's waiting areas (4.30-5.30 PM off-school period). We will put a box in the kindergarten's waiting areas for five working days (8:00 AM-5:00 PM every day) after distribution so that they can return the consent form and questionnaires. This healthcare staff will check the box every day and collect the returned consent form and questionnaires. Potential participants will have over 24 hours to decide if they want to participate.

Note: Kindergarten teachers are responsible for preschool children in each class and have regular contact with parents of preschool children. Kindergarten healthcare/nursery staff are usually responsible for child nutrition and health in each class or the whole kindergarten (e.g., meals, weight and height assessment, and safety) and have no direct contact with parents.
After receiving ethical approval from King's, this study will be registered in the local maternal and child health hospital. After registration, two HCPs in the department of child health who are our co-researchers will be responsible for delivering the intervention program. Since they will be involved in the process evaluation, JW will provide them with PIS and consent forms for semi-structured interviews.

C5 Do you have a current or prior relationship with any potential participants? (This includes professional and/or personal relationships)

- (selected) Yes, I do have a current or prior relationships with potential participants.
- ( ) No, I do not have any current or prior relationships with potential participants.

C5a If you are in a position of influence or authority over potential participants, can you confirm that this could not give rise to a perceived pressure to participate?
Please note: If you are directly involved in the teaching or assessing of participants this is considered a perceived pressure to participate.

- (selected) Yes, there is potential for my existing relationship to give rise to a perceived pressure to participate.
- No, there is no potential for my existing relationship to give rise to a perceived pressure to participate.

C5b Please outline the existing relationship(s) and explain how you will mitigate any potential pressure to participate.

Two HCPs who are our co-researchers and deliver the intervention will be asked to participate in the semi-structured interview for process evaluation. I will let them know this participation is completely voluntary and non-participation will not disadvantage them and their child (e.g., education, care) in any way.

The gatekeeper will not approach participants in person and not know who the participants are in the whole process. We make it clear in the take-home letters that their participation is completely voluntary and non-participation will not disadvantage in any way.

C6 Gatekeeper Permission: Will you require an individual or organisation to grant you permission to approach/ access your intended participants? This includes gatekeepers contacting participants on your behalf

- (selected) Yes, I will be using a gatekeeper to access potential participants
- No, I will not be using a gatekeeper to access potential participants

C6a Will the gatekeeper be in a position of influence or authority over the participants?

- (selected) Yes, the gatekeeper is in a position of influence or authority over participants
- No, the gatekeeper will not be in a position of influence or authority over participants

C6a i) Will the gatekeeper be aware of who has agreed to take part in the project?

- Yes, the gatekeeper will be aware of who participates
- (selected) No, the gatekeeper will not be aware of who participates

C6b Outline who the gatekeeper is and how they will be used to facilitate recruitment.
Please note: Participants must only be approached once appropriate gatekeeper permission has been obtained.

The gatekeeper is each head of the kindergarten from two kindergartens in Yangzhou, China.
First, we will obtain permission from each gatekeeper at two kindergartens to display the study invitation posters on notice boards and waiting areas. We will also ask three kindergarten healthcare staff to distribute the take-home letters, participant information sheet (PIS) and ineligibility statement at the most recent parent-teacher conference.

Second, we will request permission to recruit three kindergarten healthcare staff who do not have regular contact with parents. These healthcare staff will make an announcement for this study and distribute the take-home letters, participant information sheet (PIS) and ineligibility statement in the parent meeting. They will also announce that participation is completely voluntary and non-participation will not disadvantage them and their child (e.g., education, care) in any way. One of these healthcare staff will be responsible for distributing and collecting consent forms, baseline and follow-up questionnaires (blindness for group allocation). The second healthcare staff will assess these preschool children weight and height at each time point based on the student ID on the baseline questionnaire provided by their parents after obtaining the participants' consent form (blindness for group allocation). The third healthcare staff will send text messages of child's actual weight status to parents in the intervention (not blindness for group allocation). This work is voluntary because it will take place during their usual working hours. We will let kindergarten healthcare staff know that it is not mandatory to distribute/collect questionnaires and measure child weight and height. If they are unavaible to support the project, it will not affect them in any way.

Next, we will ask permission to place a box in the kindergarten's waiting areas for five working days (8.00 AM-5.00 PM every day) three times after the kindergarten healthcare staff distribute the consent form and baseline questionnaires (the first time), questionnaires/survey immediately after intervention (the second time) and questionnaires one-month follow-up (the third time) within a regular date (around two working days each time) at the kindergarten's waiting areas (4.30-5.30 PM off-school period) so that parents can return the consent form and questionnaires. This healthcare staff will check the box every day and collect the returned consent form and questionnaires.

Finally, we will request permission from gatekeepers at kindergartens to book/provide a kindergarten conference room/other available premise for intervention modules and semi-structured interviews (process evaluation).
The gatekeeper will not be aware of who participates in the whole process.
-We will also obtain permission from the parents of children that the kindergarten healthcare staff will assess their child's height and weight at each time point via the student ID they write on the questionnaire after receiving their consent form.

C6c Please outline how gatekeeper permission will be obtained

This study has received ethical approval from the local maternal and child health hospital in Yangzhou, China, and will be registered after getting approval from King's. This hospital takes the responsibility of providing guidance and checking the work related to child health in the kindergartens in Yangzhou. After registration, the staff at the hospital will communicate officially with each head of the kindergarten (gatekeeper) at two kindergartens about the research. The principal investigator (JW) will book an appointment for a face-to-face discussion of the research procedures, aims, and potential benefits with each head of the kindergarten.

C7 Please specify any incentives being offered and a justification for their use.

All participants in the intervention group will receive a gift (e.g., child plate, books, balloon, and stickers) upon completion of each module to further support their behavior change and thank them for their time. Parents involved in the control group will receive the complete material package of EPO Feeding program (handouts and supplementary materials), their child's weight status measured at the final time point, and provided access to pre-recorded modules by healthcare professionals as an incentive.
JW will provide one public lecture related to child nutrition guidelines, eating, and feeding after completing the whole project (i.e., qualitative interviews, cross-sectional study, and feasibility trial) (no later than 30/09/2024). Only parents who agree to participate in this project will have access to the public lecture and will be asked to directly email JW to sign up with their identifiable information (e.g., student ID number).
Note: all identifiable/pseudonymized information will no longer be stored by the submission deadline of JW's thesis (Expected submission date: 30/09/2024).

Section C: Informed Consent

C8 Will informed consent be sought from all participants? ◉ Yes ○ No

C8a How will informed consent be obtained for each data collection method/participant group? Who will take consent and how will it be recorded?

Note: Justification must be provided if you will not be providing all participants with an information sheet and gaining written consent. ***Please see the guidance icon for further information on tailoring informed consent processes and current templates.***

Informed consent will be obtained from all participants.
-Parents
The study informed consent process will begin during the recruitment process. If the participants are interested in participating, the principal investigator (JW) will further screen the potential participants and then give each eligible participant a consent form for review via WeChat/email. The Participants will be asked to read it and determine whether they wish to participate and proceed to sign the consent form as reported in C4.
-HCPs
The study informed consent process will begin during the recruitment process. These two HCPs will be provided with PIS and consent form via WeChat/email. JW will give them the hard copy of the consent form in person (in the local maternal and child health hospital) if they agree to sign it.

C9 How long will participants be given to decide if they wish to participate?

Please provide justification if participants will be given less than 24 hours

Parents who are interested in this study can contact the principal researcher (JW) via email/WeChat/phone call (provided on the poster, PIS and take-home letters) for further information. Potential participants contacting JW will be screened for eligibility and if eligible, they will be provided with the information sheet and consent form for their review via email/WeChat. JW will also let them know if they agree to attend, they should first complete the hard copy of the consent form and baseline questionnaire which can be collected from a healthcare staff within a regular date (around two working days) in the kindergarten’s waiting areas (4.30-5.30 PM off-school period). We will put a box in the kindergarten’s waiting areas for five working days (8:00 AM-5:00 PM every day) after distribution so that the participants can return the consent form and questionnaires. Potential participants will have enough time (at least two working days) to decide if they want to participate.

HCPs who are responsible for delivering the program will be provided with PIS and consent form via WeChat/email and informed that they have at least two working days to decide if they want to participate. If they have any questions, they can contact JW for inquiries. If they decide to take part, JW will give them a consent form to sign in person (in the local maternal and child health hospital) and will give them a countersigned copy of this consent form to keep.

C10 Detail the process by which participants may withdraw from the research both during the research and after it has been completed. A final withdrawal date should also be provided, after which participants may no longer withdraw their data from the study.

Participants can withdraw their data before a one-month follow-up assessment (around 24/02/2024).
-Parents will be able to reach the principal investigator (JW) for withdrawal requests with their child's student ID through the email provided at the bottom of the information sheet. JW will remove their data based on their child's student ID number.
- Two HCPs are also able to reach JW through email/WeChat up to one month after delivering the intervention (around 24/02/2024) to withdraw their data (i.e., semi-structured interviews).

Section D: High Risk Research

D1d Risk Identified: The study could induce psychological stress or anxiety, or produce humiliation or cause harm or negative consequences beyond the risks encountered in normal life.

i) Explain how the nature of the research could induce psychological stress or anxiety, or produce humiliation or cause harm or negative consequences beyond the risks encountered in normal life.

1) Due to the nature of the randomized process, some participants who really want to attend the intervention program may feel stressed/upset if they are allocated to the control group.
2) Some questions in the questionnaires (baseline and follow-up assessment) are related to child weight, eating, and parental feeding, which may be a little sensitive for some participants and make them feel stressed/anxious. For example, the assessment questionnaire has the questions related to their concern about child weight such as “How much you concern about your child underweight/overweight?”; and questions related to their parenting sense of competence such as “Sometimes I feel like I’m not getting anything done”, “Being a parent makes me tense and anxious”.
3) Since the intervention program takes every week and lasts 4 weeks, some participants may have the inconvenience of participating in all the modules.

ii) How will you mitigate any potential risks that may arise from the sensitive nature of the research?

1) Selection bias will be minimized by ensuring all participants eligible and recruited have equal opportunity of being allocated to each study group and follow-up completed. We will provide all related materials for parents in the control group, including access to pre-recorded presentations for four modules, handouts, supplementary materials, and their child's actual weight status at the final follow-up assessment.
2) The participants will be informed by the principal investigator (JW) before their participation of a possible risk of distress or anxiety as indicated in the information sheets. This makes them familiar with the assessment questionnaire. Participants will also be informed that if they feel uncomfortable with any of the questions, they can withdraw without any reason. If they feel upset at any stage, feel free to contact JW via WeChat/email/phone call to get counseling support. The contact details of the principal researcher (JW) also is provided in the participant information sheet, and participants can make contacts for any support.
3) To minimize the inconvenience, every effort will be made to arrange the modules. Each module will take place twice each week, so parents in the intervention can choose to attend on Friday (12.30-13.30 PM) or Saturday (09.00-10.00 AM) in the kindergarten premises at their convenience.

iii) Outline your procedure in the event of a participant becoming distressed and/or requiring additional support as a result of participation.

If a participant feels distressed or upset during the trial, they can withdraw at any time and they can speak with JW about their concerns. JW will talk with the participants about their sources of support and suggest what could be done next. The participants will be offered free, counselling, or psychological support from a psychologist at our registered maternal and child health hospital if they want. In addition, we will provide information on the support services for participants via email/WeChat: If participating in the trial has raised any concerns about own mental health, please check out Chinese psychology webpages for suggestions on support and advice: https://www.psy.com.cn/, https://ncmhc.org.cn/. Furthermore, for more information surrounding mental health, please visit https://ncmhc.org.cn/channel/newsinfo.

D2 If there are any additional risks or burdens to participants that have not been addressed above, please provide further details and explain how these risks will be mitigated:

There are no other perceived risks to participants. However, participants will be reassured that their participation is entirely voluntary and that they can choose to withdraw at any stage of the study without being obliged to give a reason before one-month follow-up assessment (detailed withdrawal instructions on information sheets).

D3 What are the potential benefits to the participant?

This information will then be used to further refine the intervention which can support Chinese parents of preschool children in improving feeding practices and accurate perception of their child's weight.
All participants in the intervention group will receive a gift (e.g., child plate, books, balloon, and stickers) upon completion of each module to further support their positive behavior change and thank them for their time.
After the final data collection at one-month follow-up, participants from the control group will be offered the complete material package of the EPO Feeding program, their child's weight status measured at the final time point, and provided access to pre-recorded modules by healthcare professionals as an incentive.
JW will provide one public lecture related to child nutrition guidelines, eating, and feeding after completing the whole project (i.e., qualitative interviews, cross-sectional study, and feasibility trial) (no later than 30/09/2024). Only parents who agree to participate in this project will have access to the public lecture. Parents who agree to participate in this project will be asked to directly email JW to sign up with their identifiable information (e.g., child student ID number).
Note: all identifiable/pseudonymized information will no longer be stored by the submission deadline of JW's thesis (Expected submission date: 30/09/2024).

We will share our final report with HCPs. They can use these materials in their speaking/lectures noted with the source of materials.

D4 Will participants be guaranteed complete anonymity in the final report and any further research output/s? (●) Yes ( ) No

D4a Please explain how you will ensure participants remain completely anonymous in the final report or any other research output/s.

Data will be processed under the terms of UK data protection law (including the UK General Data Protection Regulation (UK GDPR) and the Data Protection Act 2018). King's College London (KCL) is the sponsor for this study. The research team will be using information from the participants in order to undertake this study and will act as the data controller for this study. This means that the researcher is responsible for looking after the participants' information and using it properly.

After completing this trial, all the identifying information (i.e., preschool child student ID number and participants' contact details) will be removed. During the data analysis, only pseudonymized data will be shared with research team members. The data will no longer be identifiable by anyone after JW's thesis submission. The pseudonymized data (information from this study) will be retained for 7 years according to the KCL regulation.

All information collected during the study will be kept strictly confidential and will be stored securely and handled according to data protection guidelines. Any information about you and your child will have personal details (i.e., child student ID number and contact details) removed when shared within the research team so that no one can be recognized from it. Regarding the child's weight and height records, JW will use software to calculate child's BMI-Z score accordingly and then share BMI Z-score with the research team. Likewise, other information which may be identified will be deidentified by JW and then be shared within the research team.
Only JW will have access to the identifiable information and the entire research data. Personal information (i.e., preschool child student ID and contact detail) will be kept separately from depersonalized data, and field and kept in a locked cabinet in JW's office at King's College London. This cabinet will be situated in a locked room, for which only JW has a key. While still in China for data collection, all hard copies of documents and data will be stored in a locked cabinet in JW's place of residence at Yangzhou –Jiangsu, China, and only JW will have a key to the cabinet.

Research data will also be stored electronically and kept in an encrypted and password-protected computer, accessed only by JW. All data will be kept with the strictest confidence and participants will not individually be identifiable in all research output. Any backup (copies) of data will be held in an encrypted and password-protected external hard drive and kept in a locked cabinet accessible to JW only.

Section E

E1 Does the project involve the collection and/or use of personally identifiable information (as outlined in UK GDPR)?

Identifiable information is data that can be used to identify an individual, either directly (such as full name, address, Twitter handle, etc) or indirectly through the combination of several pieces of data. The most common examples are names, contact details, audio/ video recordings, usernames etc. However, data that has the potential to indirectly identify a participant should also be treated as identifiable.

Please see the guidance icon for more examples of when data should be considered identifiable or contact the Research Governance Office: rgo@kcl.ac.uk

Please indicate which of the following applies:

- (●) Yes, the project involves the collection and/or use of identifiable information
- ( ) No, I will not be collecting and/or accessing any identifiable information for this project.

***Before completing the following questions, please ensure you have read the KCL Research Data Management Guidelines* and guidance on the UK General Data Protection Regulation (UK GDPR)**

Section E (I): UK Data Protection Requirements

E2 Who is the Data Controller? Please see the guidance icon for a definition of a Data Controller

- (●) King's College London
- ( ) An External Individual/ Institution
- ( ) King's College London is a Joint Data Controller with an External Individual/ Institution

E3 Please state which of the following categories of personal data (relating to research participants) will be collected, processed or stored ___________ of the research project? (please select all that apply):

- [x] Name and/or signature (this includes those recorded on consent forms)
- [x] Date of Birth/ Age
- [x] Contact Details (email address, phone number, etc)
- [x] Identification Number (participant number, NHS number, staff number)
- [ ] Location Data (full address, postcode, IP address etc)
- [ ] Online Identifier (identifiers provided by devices or apps, cookies etc)
- [x] Identifiable Image or Recording (photographs, video recordings and audio recordings) including interview recordings
- [x] Biographical Data (includes gender, marital status, employment history/job, etc.)
- [ ] Other

E4 Will any of the following special categories of personal data (relating to research participants) be collected, processed or stored at any stage of the research project from this point forward? (please select all that apply):

- [ ] Race and/or Ethnic origin
- [ ] Political opinions
- [ ] Religious or philosophical beliefs
- [ ] Trade Union Membership
- [ ] Processing of genetic data
- [ ] Biometric data for the purpose of uniquely identifying a natural person
- [x] Health data
- [ ] Sex life
- [ ] Sexual orientation
- [ ] Criminal convictions or offences
- [ ] None of the above

E5 The UK GDPR identifies research as a 'public task' and as such you are advised to use 'public task' as your lawful basis for processing personal data. As you will also be collecting special category data, you are also required to state a condition for processing this data. As a KCL researcher, you are advised to use the 'archiving, research and statistics' condition for processing special category data.

Please confirm you will be processing personal and special category data under the 'Public task' and 'Archiving, research and statistics' lawful bases

- (•) Yes, I will be processing personal data under the 'Public task' and special category data under the 'archiving, research and statistics' condition for processing
- ( ) No, I will be processing data under an alternative lawful basis and/or condition for processing

Section E (II) Data Handling, Protection and Storage during data collection & analysis

E6 In which format/s will the personal data be stored in while data collection and analysis is _______?

- [x] Electronic Format
- [x] Hard Copy

E6a Electronic format - select all that apply:

Please note, where possible a KCL storage option should be selected in addition to any external storage

- [ ] KCL network drive
- [x] KCL SharePoint
- [ ] NHS Network Drive
- [x] KCL OneDrive
- [x] External hard drive
- [ ] USB
- [x] KCL laptop
- [ ] Personal laptop
- [ ] Other

E6a i) (•) I confirm that the storage device I will use is password protected and, where possible, encrypted.

E6b Hard copy - select all that apply:

- [x] Stored securely within the College
- [ ] Secure repository when in the field
- [ ] Stored securely on NHS premises
- [x] Other

Please provide details of the specific location:

At JW's office. 1.32 James Clerk Maxwell Building, Florence Nightingale Faculty of Nursing and Midwifery, King's College London, 57 Waterloo Road, London, SE1 8WA.
While still in China for this research data collection, all hard copies of documents and data will be stored in a locked cabinet in the JW’s place of residence at Yang Zhou–China, and only the principal investigator (JW) will have a key to the cabinet.

E7 Data Access: **During data collection & analysis**, will data be shared with any researcher or individual ____________________ ___________? (*Please note this includes sharing any audio/ video recordings with transcription services)*

- (•) No, data will not be shared with any other researcher or individual outside of the immediate research team during data collection and analysis
- ( ) Yes, data will be shared with another researcher or individual outside of the immediate research team during data collection and analysis (for example, for the purposes of transcribing the data)

E8 Once **data analysis is complete** how will research data (including any participant contact details) be stored:

- ( ) Data will be stored in an identifiable format after analysis
- (•) Data will be pseudonymised after analysis
- ( ) Data will be fully anonymised immediately after analysis

E8a Please outline how you will pseudonymise each category of personal data as selected under E3 & E4.

Data will be processed under the terms of UK data protection law (including the UK General Data Protection Regulation (UK GDPR) and the Data Protection Act 2018). King's College London (KCL) is the sponsor for this study. The research team will be using information from the participants in order to undertake this study and will act as the data controller for this study. This means that the researcher is responsible for looking after the participants' information and using it properly.

After completing this trial, all the identifying information (i.e., preschool child student ID number and participants' contact details) will be removed. During the data analysis, only pseudonymized data will be shared with research team members. The data will no longer be identifiable by anyone after JW's thesis submission. The personal/identifiable data will be deleted once JW's PhD thesis is submitted. The anonymous data (information from this study) will be retained for 7 years according to the KCL regulation.

All information collected during the study will be kept strictly confidential and will be stored securely and handled according to data protection guidelines. Any information about you and your child will have personal details (i.e., child ID and contact details) removed when shared within the research team so that no one can be recognized from it. Regarding child's weight and height records, JW will use software to calculate child's BMI-Z score accordingly and then share BMI Z-score with the research team. Likewise, other information that may be identified will be de-identified by JW and then be shared with the research team.

Only JW will have access to the identifiable information and the entire research data before thesis submission. Personal information (i.e., preschool child student ID and contact detail) will be kept separately from depersonalized data, and field and kept in a locked cabinet in JW's office at King's College London. This cabinet will be situated in a locked room, for which only JW has a key. While still in China for data collection, all hard copies of documents and data will be stored in a locked cabinet in JW's place of residence at Yangzhou –Jiangsu, China, and only JW will have a key to the cabinet.

Research data will also be stored electronically and kept in an encrypted and password-protected computer, accessed only by JW. All data will be kept with the strictest confidence and participants will not individually be identifiable in all research output. Any backup (copies) of data will be held in an encrypted and password-protected external hard drive and kept in a locked cabinet accessible to the principal investigator (JW) only.

Section E (III) Data Handling, Protection and Storage on completion of the research

E9 In which format/s will the personal data be stored following __________ of data collection and analysis?

- [x] Electronic Format
- [x] Hard Copy

E9a Electronic format - select all that apply:

Please note, where possible a KCL storage option should be selected in addition to any external storage

- [ ] KCL network drive
- [x] KCL SharePoint
- [ ] NHS Network Drive
- [x] KCL OneDrive
- [x] External hard drive
- [ ] USB
- [x] KCL laptop
- [ ] Personal laptop
- [ ] Other

E9a i) (•) I confirm that the storage device I will use is password protected and, where possible, encrypted.

E9b Hard copy - select all that apply:

- [x] Stored securely within the College
- [ ] Stored securely on NHS premises
- [x] Other

Please provide details of the specific location:

At JW's office. 1.32 James Clerk Maxwell Building, Florence Nightingale Faculty of Nursing and Midwifery, King's College London, 57 Waterloo Road, London, SE1 8WA.

While still in China for this research data collection, all hard copies of documents and data will be stored in a locked cabinet in JW's place of residence at Yang Zhou–China, and only the principal investigator (JW) will have a key to the cabinet.

E10 Expected date that the data (including any participant contact details) will no longer be stored in an identifiable/pseudonymised format:

Please note: Data should only be stored in an identifiable format for as long as is absolutely necessary.

30/09/2024

E11 Data Retention Schedule

Research data should be stored in line with the KCL Data Retention Schedule. Please note that raw data should be stored in an ***anonymous/ pseudonymous*** format where possible.

- [x] I confirm that research data will be stored in line with the KCL Data Retention Schedule

E12 Data Access: Please confirm that no other researcher or individual outside of the immediate research team will have access to any personal data on completion of data collection and analysis

- (●) Yes, I confirm that no other researcher or individual outside of the immediate research team will have access to any personal data on completion of data collection and analysis
- ( ) No, another researcher or individual outside of the immediate research team will have access to personal data on completion of data collection and analysis

Section E (IV): Publication & Data Sharing on completion of the research

E13a Will any data from which participants could be identified be published (this could be direct quotes or biographical data that could lead to the identification of an individual)?

- ( ) Yes
- (●) No

E14 Will research data be shared with any external third parties **after data analysis is complete**?

- ○ Yes
- ● No

E15 Will data be archived for further use?

- ● Yes
- ○ No

E15a Will the archived data contain identifiable information?

- ○ Yes
- ● No

E16 Research Dissemination: How will results be disseminated?

- [x] Internal report (dissertation/thesis)
- [x] Journals
- [x] Conference
- [ ] Other

Section H: Insurance, Risks and Ethical Issues

H1 Does the project involve any of the Risk Assessment criteria outlined in the information icon guidance? ○ Yes ● No

H2 Project Insurance Cover - Please indicate if your project involves any of the following -

- [ ] An overseas clinical trial
- [ ] Recruitment of overseas healthcare patients
- [ ] A physical or mental health intervention involving human subjects (see guidance icon for definition)
- [x] None of the above

Please note: *As your study does not involve any of the above, we can confirm that your study would be covered under the College's current insurance policies, subject to the relevant policy terms and conditions.*

Please note that should there be any change to your study which changes your answer to question H2 above, then please contact Tania Pattenden (tel: 0207 848 3281 or e-mail: tania.pattenden@kcl.ac.uk) so that we can ensure appropriate insurance cover can be placed via consultation with our brokers. You will also need to submit a REMAS modification request to update your ethics protocol.

H3 Travel Insurance for overseas studies: I confirm that my travel insurance arrangements are as follows:

- ○ a) I will secure College travel insurance (see guidance icon for further details)
- ○ b) I will secure personal travel insurance
- ● c) I do not require travel insurance as I will conduct the research in my country of legal residence
- ○ d) I will not secure travel insurance for overseas travel

H4 I confirm that if Disclosure & Barring Service clearance (or overseas equivalent if conducting research abroad) is required for my study, this will be obtained prior to the commencement of data collection.

(•) Yes ( ) No ( ) N/A
