# Supplementary figures and images for: Empowering parents to optimize feeding practices with preschool children (EPO-Feeding): A study protocol for a feasibility randomized controlled trial
Source: PLoS One. 2024 Jun 3;19(6):e0304707. doi: 10.1371/journal.pone.0304707 (PMC11146728; doi:10.1371/journal.pone.0304707)

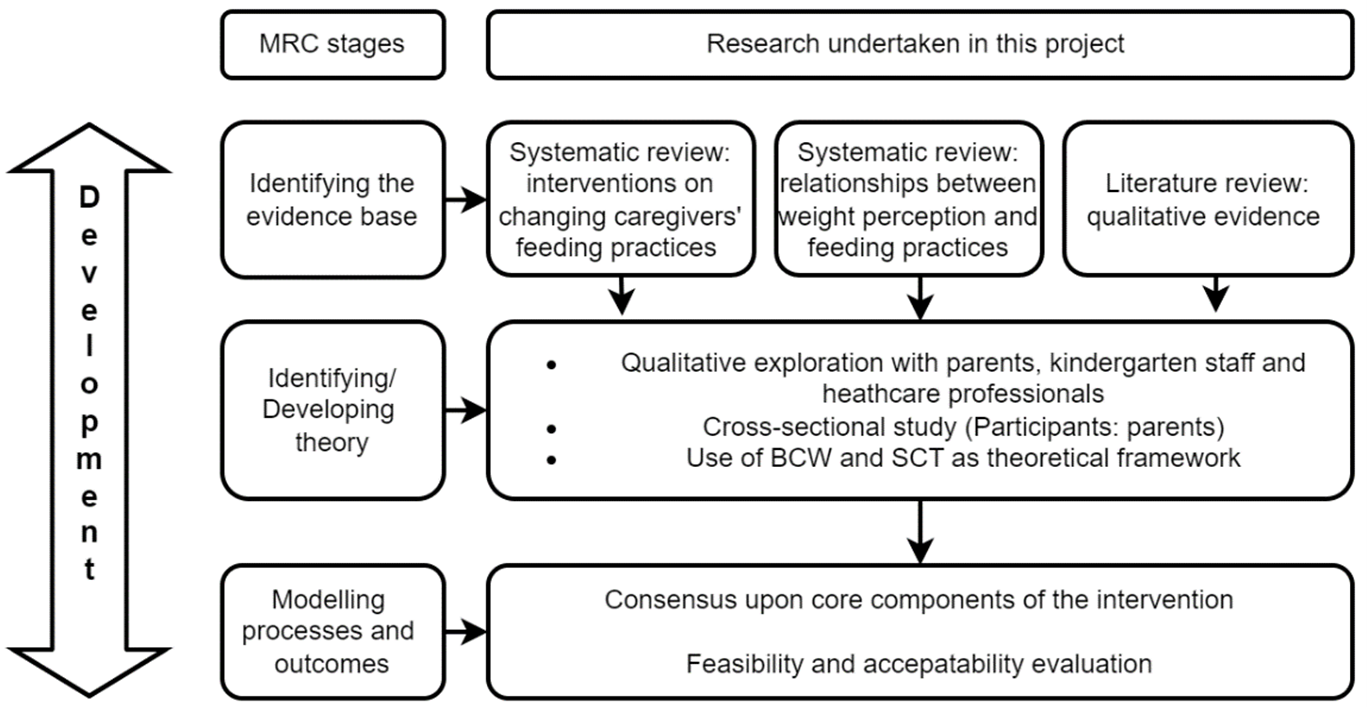

Supplement: S1 Fig — (TIF) [file pone.0304707.s003.tif]
