# Supplementary material for: Empowering parents to optimize feeding practices with preschool children (EPO-Feeding): A study protocol for a feasibility randomized controlled trial
Source: PLoS One. 2024 Jun 3;19(6):e0304707. doi: 10.1371/journal.pone.0304707 (PMC11146728; doi:10.1371/journal.pone.0304707)
Supplement: S1 Table — (DOCX) [file pone.0304707.s004.docx]

**S1 Table. Indicative topic guide for EPO-Feeding program**

| **Participants (Parents)** | |
| --- | --- |
| Acceptability and feasibility | How would you describe your experience of participating in this training program? |
|  | How do you value EPO-Feeding Program in your daily feeding practice? Any indicative changes in your behaviours? |
|  | How would you describe your experience of supporting your preschool child eating behaviours through EPO-Feeding Program? |
| Satisfaction | How satisfied are you with each/overall training module? |
|  | How clear do you find the objectives, ideas, and content in the module? |
| Suggestions for further  improvement | What do you think could be improved in each module? Is there anything missing? |
|  | What do you least like about the program? How can they be resolved in your view? |
| Barriers and enablers | Are there any difficulties in taking part? |
|  | What are the barriers/enablers to completing these modules, and applying your learning to daily life? |
|  | What are the challenges of completing the assessment, with questions relating to reasons for not taking part/ discontinuation or dropping out? |
| **Healthcare professionals** | |
| Acceptability and feasibility | What do you think of EPO-Feeding Program? |
|  | What participants thought about this program? Which parts worked, which parts did not work? |
| Satisfaction | How satisfied are you with each/overall module (including adaptation and development)? |
| Suggestions for further improvement | What do you least like about the program? What do you think could be improved? Is there anything missing? |
| Barriers and enablers | Are there any difficulties in delivering the program? |
|  | What are the barriers/enablers to deliver modules? |
