# Supplementary material for: Empowering parents to optimize feeding practices with preschool children (EPO-Feeding): A study protocol for a feasibility randomized controlled trial
Source: PLoS One. 2024 Jun 3;19(6):e0304707. doi: 10.1371/journal.pone.0304707 (PMC11146728; doi:10.1371/journal.pone.0304707)
Supplement: S2 Table — (DOCX) [file pone.0304707.s005.docx]

**S2 Table. Observation checklist of EPO-Feeding program**

| To what standard is the core content being delivered? |
| --- |
| Are the standard manual and resources being used? |
| To what extent are the key learning outcomes of each module achieved? |
| Rate the healthcare professionals |
| 1) A variety of approaches to each module using facilitation, presentation, group discussion, homework activities, feedback |
| 2) Engagement/rapport with group |
| 3) Group management/involvement |
| 4) Challenge negative behaviour within group |
| 5) Positively addressed resistance to program |
| Additional comments: |
