# Supplementary material for: Empowering parents to optimize feeding practices with preschool children (EPO-Feeding): A study protocol for a feasibility randomized controlled trial
Source: PLoS One. 2024 Jun 3;19(6):e0304707. doi: 10.1371/journal.pone.0304707 (PMC11146728; doi:10.1371/journal.pone.0304707)
Supplement: S3 Table — (DOCX) [file pone.0304707.s006.docx]

**S3 Table. Fidelity checklist of the EPO-Feeding program delivery**

| **Domain** | **Assessment method** | **Quality criteria** |
| --- | --- | --- |
| Intervention program design | Assess whether the EPO-Feeding program manual reflects the underlying theoretical model and the findings from the systematic reviews, qualitative study, and cross-sectional study | 1. Before study implementation, investigators and optimally a protocol advisory committee (three supervisors, two HCPs and two kindergarten teachers) should review the EPO-Feeding Program manual to ensure that the active/core ingredients are fully operationalized. 2. The extent to which the components reflect the hypothesized theoretical constructs and mechanisms of action should be assessed. |
| Healthcare professionals | Assess their knowledge and skills acquisition | 1. Ensure providers are trained to a well-defined performance and good understanding of each module that they are responsible for (e.g., slides, handouts, and pre-recording of the module). 2. Trainers should have professional knowledge and skills in child feeding, nutrition, and development (e.g., dietitians, healthcare professionals, and kindergarten healthcare teachers). |
| EPO-Feeding Program modules | 1. Ask the extent participants understand the intervention through qualitative interviews/motivational interviewing. 2. Assess the participant's ability and willingness to adopt these knowledge and skills | 1. Verify the participants’ understanding of the modules provided (via interviews). 2. Evaluate if they can use the skills and recommendations (via homework activities, interviews). |
