# Supplementary material for: Empowering parents to optimize feeding practices with preschool children (EPO-Feeding): A study protocol for a feasibility randomized controlled trial
Source: PLoS One. 2024 Jun 3;19(6):e0304707. doi: 10.1371/journal.pone.0304707 (PMC11146728; doi:10.1371/journal.pone.0304707)
Supplement: Attachment — Submitted filename: Response to Reviewers.docx [file pone.0304707.s007.docx]

Dear Editors and Reviewers,

Thank you very much for reviewing our manuscript “Empowering parents to optimize feeding practices with preschool children (EPO-Feeding): A study protocol for a feasibility randomized controlled trial” **(ID: PONE-D-24-05284)**. We greatly appreciate the constructive feedback from your reviewers and editorial staff. Your advice helped us improve this manuscript. Please forward our heartfelt thanks to these experts.

Based on your comments, we revised our manuscript and explained the revisions after each comment. All changes within the manuscript are highlighted using the track changes feature in MS Word. **The line numbers in this response letter correspond to those in the resubmitted manuscript with the track changes.**

We hope the changes are satisfactory. We look forward to hearing from you regarding our submission. We would be glad to respond to any further questions and comments you may have.

Yours sincerely

**Editor comments:**

The reviewers point to minor points to expand on, or add, for the introductions and methods, including more information on prior responsive feeding studies, incidence of obesity and overweight in China for this age group (reviewer 1), as well as of feeding practices in the centers (reviewer 3), additional considerations for inclusion/exclusion (reviewer 3), more detail on the intervention and content (see reviewer 1 and 3), information on validity and reliability (see comments by reviewers 1 & 2), and on the qualitative study.

For analyses, see comments by reviewer 1 on qualitative analyses, as well as by reviewer 2 in the presence of non-normality, for missing data, and for linear mixed-effect regression analyses.

We appreciate your thoughtful summary of the reviewer's comments. This has given us a valuable and constructive perspective to guide our manuscript revisions. We have made the revisions as outlined below:

(1) *Introduction section*

- We have added more details and findings of our recent systematic review that included prior intervention studies on improving caregivers’ feeding practices (Lines 82-92).
- We have also presented the prevalence of childhood overweight and obesity in China for this age group (Lines 51-53).
- In addition, we have provided clarification and specification of our intervention context (i.e., Chinese family-based food environment) (Lines 54-62) and given more explanation to address the comment from reviewer 3 regarding considering the kindergarten context (Page 17 in this response letter).

(2) *Methods*

- We have clarified one of the inclusion criteria (i.e., Parents with at least one preschool child aged 2 to 6 years enrolled in kindergarten…) to address the comments from reviewer 3 (i.e., the consideration of kindergarten food context) (Line 173, Table 1).
- We have also included the content and components of our EPO-Feeding program (Line 281, Table 2) and reported it following TIDieR checklist [1] (comments from reviewers 1& 3). Furthermore, we added information on the reliability and validity of the included questionnaires (comments by reviewers 1 & 2) (Lines 330-335, 337-343, 346-354, 358-362).
- Regarding the qualitative study (i.e., process evaluation), we have added more details on its design, sampling, recruitment, and analysis (Lines 378-383, 388-403; Section Process evaluation).

(3) *Analyses*

- Based on the comments from reviewers 1 & 2, we have revised the statistical analysis for testing the effects of the EPO-Feeding program (i.e., secondary outcome) and added more details on the (progression) criteria for the feasibility and acceptability related to the missing data (Lines 420-428; Section Data analysis plan).

In addition to the comments by reviewers, please address the following edits:

Line 165. Who is XXW? Should this be XW according to the cover page? Same for lines 372 and 378.

Thank you. We’re sorry for this mistake. We have corrected the abbreviation of co-author Xiaoxue Wei's name from 'XXW' to 'XW' in the paper.

Line 170. Unmasking is needed for analyses. Should this say “Unmasking will not take place until the databases are closed, and the data collection has been finished.”?

Thank you. Your suggestion accurately clarifies the timing of unmasking. We’ve updated the statement accordingly.

“Unmasking will not take place until the databases are closed, and the data collection is finished.” (Line 194)

**Reviewer** **comments:**
**Reviewer #1**: Dietary choices and feeding behaviours are shaped early in life; however, few intervention studies investigate the effectiveness of parenting guidance and support on early feeding. The authors have developed an evidence-informed intervention (based on a review of the literature and formative interviews), which they now propose testing for feasibility and acceptance prior to implementing a larger scale efficacy trial. Overall, the protocol is presented clearly and aligns with the trial registration information documented in ClinicTrials.Gov. My comments are intended to further clarify the design and future analyses:

Introduction

- The introduction justifies the need for the intervention study. However, I would have liked to have seen more information from prior intervention studies on the promotion of responsive feeding to understand better the number of interventions implemented, effectiveness on feeding behaviours, and limitations in the evidence.

We appreciate your comment. We’ve added more relevant information as follows.

“Despite this evidence, our recent systematic review included eighteen studies (i.e., thirteen randomized controlled trials (RCTs) and five non-RCTs) with eighteen intervention programs to test the effectiveness of interventions on optimizing caregivers’ feeding practices with preschool children [2]. The results indicated the inconsistent effects of the existing interventions on feeding practices, with many included studies reporting non-significant effects [2-6]. The absence of intervention effects may be attributed to limited studies prioritizing feeding practices as primary outcomes and incorporating explicit content around responsive feeding. Instead, most of the included studies primarily targeted child nutritional-related issues (e.g., child obesity prevention and healthy eating promotion) [2]. Therefore, there is a need to develop intervention programs which focus on the most effective ways to optimize feeding practices.” (Lines 82-92)

- Feeding behaviours are shaped by a number of factors, including culture. The authors describe the attitudes and perceptions on feeding preschoolers in this part of China. It will also be valuable to briefly note the general status of overweight and obesity in China in this age group.

Thank you. We’ve added the relevant information as follows.

“In 2020, about 7% of Chinese children under the age of 6 were overweight and 3.6% were obese, representing the largest child population with obesity globally [7].” (Lines 51-53)

Methods

- It will be useful to include the TIDier table (Hoffmann et al 2012, BMJ) to describe the intervention content and delivery in more detail for readers in the main paper.

Thank you for this suggestion. We’ve revised S2 Table (summary of intervention) and put it in the main text (Table 2).

“……specific intervention content in each intervention topic (Line 281, Table 2), which were concluded by combining the findings from our systematic reviews, a qualitative study and a cross-sectional study and reported following the TIDieR checklist [1]” (Lines 233-236)

**Table 2 Content and components of the EPO-Feeding program**

| **Theme of module** | **Objectives** | **Description of intervention content** | **Implementation** |
| --- | --- | --- | --- |
| 0. Introduction | - Increase the awareness of feeding practices - Outline the overview of the EPO-Feeding program | *Slide shows*   - What are feeding practices? - What is EPO-Feeding program? (background, development process, aims, brief content, and context) - The detailed schedule of the program (time, location) | Location: kindergarten classroom  Provider: healthcare professionals (HCPs) |
| 1. Understand children’s growth process, nutrition, and health | - Help parents understand their preschool children’s growth process and nutritional guidelines for healthy eating - Enhance parental awareness about physical signs of the normal and abnormal child growth process and weight status - Promote parental accurate evaluation of preschool child weight status - Facilitate parental knowledge of the consequences of underweight and overweight/obesity in children | *Session (slide shows)*   - The characteristics of preschool children's growth and eating behaviours - The standard evaluation of child growth and weight status - Nutritional guidelines for preschool children's eating behaviours - The cause and consequences of childhood overweight/obesity/underweight   *Handouts*   - Growth standard for children under 7 years of age (Chinese Standard 2022) - The nutritional guideline for Chinese preschool children eating by the Chinese government/Nutrition Society   *Group discussion*   - Group discussion: Participants can discuss their estimation of children’s weight status. What do participants think of this estimation method?   *Counselling*   - Q&A: Raise questions related to child growth and development, the healthcare provider will answer their questions. - Feedback: Assess children’s weight and height at baseline, immediately after the feasibility intervention and one-month follow-up. Send text messages to let parents know the child's actual weight status by kindergarten teachers. - Recommendation: some free authoritative software/App that can help parents monitor their child's weight status and growth in daily life.   *Educational videos*   - Share video resources on how to draw children's growth curves released by Chinese public authority websites via the WeChat group. | Location: kindergarten classroom  Provider: HCPs  Theory:  BCT   - Shaping knowledge - Social support - Repetition and substitution - Natural consequences - Feedback and monitoring - Comparison of outcomes - Comparison of behaviour   SCT   - Reciprocal determinism - Outcome expectations - Self-efficacy - Observational learning - Facilitation - Reinforcement |
| 2. Keeping a meaningful parent/child role | - Identify specific parent/child roles in the family food environment - Facilitate and enhance parental awareness of a meaningful parent/child role | *Session (slide shows)*   - 5-keys to the division of responsibility in feeding. - Share a story to make parents aware of parent/child roles in daily life (based on the findings from our qualitative study). - Details on parental role /responsibilities (1.e., what/when/where food is served) - Details on child role/responsibilities (i.e., how much to eat, whether to eat or not)   *Group discussion*   - What is one thing that you are doing well right now regarding WHAT to serve? - Can you let your child decide how much to eat? What problems do you see with this?   *Setting goals and feedback*   - Setting goals for a new role/updated role - Healthcare providers help check it and provide feedback   *Handouts*   - Feeding role/responsibilities (Parent/Child) - What to feed your child | Location: kindergarten classroom  Provider: HCPs  Theory:  BCTs   - Shaping knowledge - Social support - Goals and planning - Repetition and substitution - Natural consequences - Comparison of behaviour - Feedback and monitoring   The Satter Feeding Dynamics Model |
| 3. Creating and maintaining a healthy food environment | - Instruct parents on how to create and maintain a healthy food environment - Enhance parental awareness about the importance of a healthy food environment - Reinforce parental responsibility in providing a good food environment | *Session (slide shows, handouts)*   - Family meals together - How to create great mealtimes? - The principles for food preparation - The routines for mealtime/snack - Co-parenting   *Group discussion*   - Share their family mealtime routine - What makes creating and maintaining a healthy food environment challenging for you?   *Setting goals and feedback*   - Setting goals for the routines of mealtime/snack - Healthcare providers help check it and provide feedback   *Educational videos*   - Share video resources on how to establish meal/snack routines released by Chinese public authority websites via the WeChat group | Location: kindergarten classroom  Provider: HCPs  Theory:  BCTs   - Shaping knowledge - Social support - Goals and planning - Repetition and substitution - Natural consequences - Comparison of outcomes - Comparison of behaviour |
| 4. Adopting appropriate feeding practices | - Facilitate parental knowledge of feeding practices - Enhance parental knowledge of the benefits and efficacy of responsive feeding practices in the promotion of healthy eating behaviours in preschool children - Enhance parental awareness of the negative effects of non-responsive feeding practices on child eating behaviours in preschool children - Help parents understand their children’s eating characteristics and respond to children's eating behaviours effectively | *Session (slide shows and handouts)*   - Responsive feeding practices   -Definition/Meaning  -Specific feeding practices  -Example: Modelling (what, how, where)  -Share a story (from our qualitative study)  -The consequence of adopting responsive feeding practices (positive)   - Non-responsive feeding practices   -Definition/Meaning  -Specific feeding practices  -Example: pressure to eat (what, how, where)  -Share a story (from our qualitative study)  -The consequence of adopting non-responsive feeding practices (negative)   - How to appropriately respond to child eating behaviours   -Introduce the common child eating behaviours (e.g., picky eating)  -Keep in mind your Feeding Role and Child Role (reinforcement)  -Respond to children’s eating emotions and encourage positive behaviours  *Setting goals and feedback*   - Setting goals for how to improve feeding practices - Healthcare providers help check it and provide feedback   *Group discussion and feedback (examples)*   - Which feeding practices do you usually adopt during the mealtime? Are there any changes/effects? - Which feeding practices would you like to recommend? Why? - Which feeding practices do you recommend when facing children’s eating problems?   *Educational videos*   - Share video resources related to caregivers’ appropriate feeding practices released by Chinese public authority websites via the WeChat group. | Location: kindergarten classroom  Provider: HCPs  BCTs   - Shaping knowledge - Social support - Goals and planning - Repetition and substitution - Natural consequences - Feedback and monitoring - Comparison of behaviour - Comparison of outcomes   SCT   - Outcome expectations - Self-efficacy - Observational learning - Facilitation - Reinforcement |
| Homework (share and feedback via WeChat) | *After module 1*   - Evaluate their child's weight status based on Chinese growth standard for children under 7 years of age (2022) and share the result with our research team or via WeChat. - Regularly evaluate and record child’s weight status with distributed record forms to help monitor their child’s growth, ensure proper nutrition, and identify potential health issues early.   *After module 2*   - What to do this week - fill out the distributed family feeding questionnaire to help identify what you are doing well and what needs to change to fully implement the program (self-monitoring)-Family feeding questionnaire (self-monitoring)   *After module 3*   - Participants will be asked to post photos, recipes and personal experiences and ideas that they had found helpful in creating a healthy food environment via the WeChat group. - At home: Introduce new foods according to children’s previous experiences. Promote children’s food experiences through colours (e.g., choose foods of a specific colour for a meal). - At home: Observe routines for food preparation and cooking, mealtimes, and feeding environment.   *After module 4*   - Ask parents to identify the positive outcomes and expectations from performing the responsive feeding practice (share feelings/experiences/photos/recording) via the WeChat group. - Ask parents to share their experiences/feelings when they use the non-responsive feeding practices and their children’s reactions via the WeChat group. | | |
| Follow-up facilitation | - Send four weekly infographics summarising the key points from each module via the WeChat group before the one-month follow-up. | | |
| Individual support (phone/WeChat calls and text messages) | - Motivational interviewing by healthcare providers: using the motivational interviewing technique to motivate the participants’ positive changes in feeding practices via WeChat/phone calls (about 20 minutes per participant). - Send text messages to participants about their child’s actual weight status a week before the follow-up evaluation (post-intervention and one month after intervention). | | |

The program content reporting follows the TIDieR checklist, covering theory and objectives (why), materials and procedures (what), intervention provider (who), delivery methods and locations (how and where), training schedule and intensity (when and how much), personalization and adaptations (tailoring), and adherence and fidelity (how well).

- It will be useful to include information on questionnaire reliability and validity previously reported in the Chinese versions of instruments employed (e.g., CFQ, Parenting Sense of Competence Scale)

Thank you. We’ve added more information. Please see below.

***Parental feeding practices*** “Each item of CPCFBS and C-CFQ is rated on a 5-point Likert scale. Each subscale is calculated by averaging the scores of all the items in that subscale with higher scores indicating a greater adoption of that feeding practice. Both questionnaires have been extensively used in Chinese samples [8-11], indicating good internal consistency reliability (CPCFBS: Cronbach's α = 0.73-0.90; C-CFQ: Cronbach's α = 0.75-0.89) [8, 9] and good construct validity [12, 13].” (Lines 330-335)

***Parental perception of preschool child weight*** “Parental self-reported perception of child weight is assessed using one item, “How would you describe your child's weight?”, which has been used and validated in previous studies [8, 9].” “Parental visual PCW will be assessed by the Parents’ Perception of Healthy Weight (PPHW) for children aged 2 to 6 years old [14].The use of PPHW has been supported conceptually [15] and empirically by studies on Asian populations [16, 17].” (Lines 338-343)

***Parenting Sense of Competence*** “It includes two subscales: 8 items in the efficacy measuring parental perception of competence in the parenting role and 9 items in the satisfaction subscale assessing parental satisfaction and comfort with the parenting role [18]. Each item is rated on a 6-point Likert scale, from “Absolutely disagree” to “Absolutely agree”. Each subscale is calculated by summing the scores of all the items in that subscale with higher scores indicating higher parenting competence and satisfaction. The Parenting Sense of Competence Scale indicated good internal consistency (Cronbach's α = 0.85) and test-retest reliability (intraclass correlation coefficient = 0.87) [18].” (Lines 346-354)

***Child eating behaviours*** “Each item is rated on a 5-point Likert scale with higher scores indicating a greater adoption of that eating behaviour. Each subscale is calculated by averaging the scores of all the items in that subscale. This questionnaire has been frequently used in China [8, 10, 11], indicating good internal consistency reliability (Cronbach's α = 0.70-0.79) [8] and validity [19].” (Lines 358-362)

Qualitative Study: (1) More elaboration is needed on the design (i.e., what qualitative research design will be implemented)? (2) All qualitative study samples are 'purposive'. More information on the sampling strategy; for example, will sample include all intervention arm and control arm parents, how will the parents in one (or both) arm/s be selected? (3) Will analyses be supported by software? (4) Will coding be inductive, deductive or both?

Thank you. Please see below. We’ve added detailed information below.

(1) Qualitative research design: “We will use a qualitative descriptive design to elicit the ‘who, what, and where of events or experiences’ from a subjective perspective with individual semi-structured interviews [20]. This pragmatic approach enables researchers to explore participants' experiences in context and to stay close to the data, using broad ‘free-form’ methods to describe participants' experiences [20]. The study reporting will follow the consolidated criteria for reporting qualitative research (COREQ) checklist [21].” (Lines 378-383)
(2) Sampling strategy: “The sample size will be determined based on the principle of data saturation, where data collection continues until preliminary analyses indicate that no new data with meaningful coherence are obtained [22]. Given the target qualitative sample participants, we aim to recruit 18-22 parents (i.e., those who attend all modules or a part of modules or drop out or are allocated to the control group) and two healthcare professionals who deliver the program. To better explore participants’ perspectives of the EPO-Feeding program and understand the program’s acceptability, we aim to include 12-13 participants who may complete all modules, 2-3 participants who may complete some of the modules, 2-3 participants who are allocated to the control group and 2-3 participants who may drop out completely. After completion of modules, the participants will be sent an invitation via WeChat by the researcher (JW) to participate in a semi-structured interview. If some participants drop out, the researcher (JW) will send messages to them via WeChat and then ask if they would be willing to be interviewed in person or online. If participants agree, a semi-structured interview will be conducted after receiving their written informed consent and will last 25-45 min. All interviews will be audio-recorded. Reflective field notes and memos will be taken to capture JW’s observations and insights, guiding the follow-up interviews.” (Lines 388-403)
(3) Software: “NVivo 14 will be utilized to organize data, make memos, and assist in coding and analysis.” (Line 477-478)
(4) Coding methods: “Then, JW and XW will initially code 20% of transcripts independently, using both inductive and deductive coding processes. Inductive coding involves codes that are developed organically from data, while deductive coding involves codes derived from applying prior knowledge, personal experience, and pre-existing concepts to the data.” (Lines 479-483)

**Reviewer #2**: The title is a bit too long and could be improved. The word protocol is to be added.

Thank you for this important feedback. We’ve shortened the title.

“Empowering parents to optimize feeding practices with preschool children (EPO-Feeding): A study protocol for a feasibility randomized controlled trial” (Title)

Line 158, the word minimal is to be replaced with minimum.

Thank you. We’ve changed “minimal” to “minimum”. (Line 181)

Line 268, whether an existing questionnaire or newly developed questionnaire was used is to be clearly stated. Information on reliability and validity is to be provided.

Thank you. The questionnaires used in this study were all existing and tested for reliability and validity with Chinese populations. We’ve added the relevant information as follows.

***Parental feeding practices*** “Each item of CPCFBS and C-CFQ is rated on a 5-point Likert scale. Each subscale is calculated by averaging the scores of all the items in that subscale with higher scores indicating a greater adoption of that feeding practice. Both questionnaires have been extensively used in Chinese samples [8-11], indicating good internal consistency reliability (CPCFBS: Cronbach's α = 0.73-0.90; C-CFQ: Cronbach's α = 0.75-0.89) [8, 9] and good construct validity [12, 13].” (Lines 330-335)

***Parental perception of preschool child weight*** “Parental self-reported perception of child weight is assessed using one item, “How would you describe your child's weight?”, which has been used and validated in previous studies [8, 9].” “Parental visual PCW will be assessed by the Parents’ Perception of Healthy Weight (PPHW) for children aged 2 to 6 years old [14].The use of PPHW has been supported conceptually [15] and empirically by studies on Asian populations [16, 17].” (Lines 338-343)

***Parenting Sense of Competence*** “It includes two subscales: 8 items in the efficacy measuring parental perception of competence in the parenting role and 9 items in the satisfaction subscale assessing parental satisfaction and comfort with the parenting role [18]. Each item is rated on a 6-point Likert scale, from “Absolutely disagree” to “Absolutely agree”. Each subscale is calculated by summing the scores of all the items in that subscale with higher scores indicating higher parenting competence and satisfaction. The Parenting Sense of Competence Scale indicated good internal consistency (Cronbach's α = 0.85) and test-retest reliability (intraclass correlation coefficient = 0.87) [18].” (Lines 346-354)

***Child eating behaviours*** “Each item is rated on a 5-point Likert scale with higher scores indicating a greater adoption of that eating behaviour. Each subscale is calculated by averaging the scores of all the items in that subscale. This questionnaire has been frequently used in China [8, 10, 11], indicating good internal consistency reliability (Cronbach's α = 0.70-0.79) [8] and validity [19].” (Lines 358-362)

Line 272, Line 347, the description of the pattern of missing data before the imputation method is to be described.

Thank you. We’ve outlined it as follows.

"Cases with missing data ≥10 % at each time point will be removed. An appropriate imputation method will be considered if cases with missing data < 10 %. We will first conduct Little's MCAR test to assess the randomness of the missing data [23]. If the test indicates that the missing data are missing completely at random (MCAR) (i.e., p-value in Little's MCAR ≥ 0.05), we will use simple imputation methods such as mean or median imputation. If the missingness is not completely random (i.e., p-value in Little's MCAR < 0.05), multiple imputation or maximum likelihood estimation will be applied." (Lines 442-449)

Line 299-302, spacing between the sentences is to be standardized.

Thank you. We’ve checked and revised the spacing.

Line 346, the word limit to be replaced with other word e.g. mitigate etc.

Thank you for this suggestion. We’ve changed “limit” to “mitigate”.

“Intention-to-treat (ITT) principles will be used for parametric data to mitigate attrition and analytical bias.” (Line 434)

Line 353-354, the test is employed when data are skewed. An alternative test is to be mentioned if the data does not meet normality assumptions. Line 354, an alternative statistical test is to be mentioned if the assumptions for the r x c contingency table/chi-square test are not met.

Thank you for the comments. We’ve added more relevant information.

“The baseline characteristics of participants in two groups will be compared using independent samples t-tests/one-way ANOVA (for continuous variables) and chi-squared tests (for categorical variables). In cases where the assumptions for the t-tests or chi-squared tests are violated, alternative methods, such as non-parametric tests for non-normally distributed data and Fisher's exact test will be considered, respectively[24].” (Lines 438-442)

Line 355, the sentence requires revision.

For primary outcomes, the handling of missing data (if any) is to be described. Is the method of handling missing data and analyses approach for secondary outcomes will be similar for primary outcomes? The write-up for the data analysis plans requires improvement in terms of flow and clarity.

We appreciate this important comment. Yes, it is important to report the missing data to determine the feasibility and acceptability of our program. As you suggested, we restructured the sentences for clarification and detailed the handling of missing data separately for primary and secondary outcomes. Please see below.

“The participant retention rate will be calculated as the rate of completion of a one-month follow-up. We anticipate less than 20% of enrolled participants will drop out, considering previous estimations of a dropout rate ranging from 10% to 16% in similar RCTs [3, 6, 25]. Regarding the feasibility of module retention, the criterion is that at least 80% of participants complete three out of four modules (> 80% of intervention content), consistent with previous relevant studies [4, 26]. The feasibility of measurements assessment depends on two criteria [6, 25, 27]: at least 80% of participants completing all the measurements, and at least 80% of completed measurements with missing values < 10% at each time point.” (Lines 420-428)

“Cases with missing data ≥10 % at each time point will be removed. An appropriate imputation method will be considered if the cases with missing data < 10 %. We will first conduct Little's MCAR test to assess the randomness of the missing data [23]. If the test indicates that the missing data are missing completely at random (MCAR) (i.e., p-value in Little's MCAR ≥ 0.05), we will use simple imputation methods such as mean or median imputation. If the missingness is not completely random (i.e., p-value in Little's MCAR < 0.05), multiple imputation or maximum likelihood estimation will be applied. Considering the ITT principals and completer analysis, participants included in the analysis will be those who have attended at least three modules and completed all measurements with missing values < 10% at each time point to test the effects of the EPO-Feeding program.” (Lines 442-452)

For linear mixed-effects regression analyses, the approach is not clear. Information on the time points (baseline, T1, and T2) (repeated measures), covariance structure, estimation method is to be mentioned. If there are covariates/confounding factors in the analysis, the proposed sample size may not have enough statistical power to detect the effects while controlling for these factors. This is to be considered in the discussion/limitation.

We appreciate this important feedback to help us reconsider which method should be used to analyse the effects of the intervention program (Secondary outcome). We’ve revised it and added some limitations in the Discussion section as you commented.

“We will utilize analysis of variance (ANOVA) for repeated measures to assess data collected at baseline, T1, and T2 for each participant, with time as the within-subjects and group condition (intervention vs. control group) as the between-subjects variables, to determine whether there are significant changes in continuous variables (i.e., feeding practices, parenting sense of competence and child eating behaviours) during the intervention period (i.e., time*group interaction). Appropriate corrections such as the Greenhouse-Geisser or Huynh-Feldt corrections will be applied to address potential violations of the assumption of sphericity. This approach assumes homogeneity of variances across groups and conditions, necessitates complete data for all time points per participant, and assumes linearity between the outcome variable and covariates. In cases where the criteria for ANOVA for repeated measures cannot be met, alternative statistical methods such as Generalized Estimating Equations (GEE) with an exchangeable correlation structure will be used. Additionally, GEE will be applied to compare the categorical outcome measures (i.e., parental perception of child weight status (misperception vs. non-misperception) and child weight status) across the time points between the two groups. GEE offer a robust approach for analysing correlated data, without stringent assumptions about the covariance structure and does not assume linearity between the outcome variable and covariates [28]. These models will incorporate potential confounders of the outcome variables as covariates (e.g., child age).” (Lines 452-469)

“Lastly, given that our trial primarily focused on assessing the feasibility and acceptability of the EPO-Feeding program instead of intervention effects, it is worth noting that the sample size may not have sufficient power to detect potential effects while controlling for various factors. However, the results from the current study may offer preliminary indications of the magnitude and direction of the intervention effects.” (Lines 544-549)

For the discussion section, some sentences are to be written in future tense.

Thank you. We’ve checked and revised the tense in the discussion section.

The list of references did not conform to the journal format.

Thank you. We’ve checked and changed the reference format following journal guidelines.

The numbering for each title/topic/subtitle/subtopic is to be omitted.

Thank you. We’ve omitted these numbering as you commented.

Figure 2, the dark background and white font are to be replaced with white and dark respectively.

Thank you. We’ve uploaded the updated Figure 2 as you suggested.

Figure 2. Participant flowchart

**Reviewer #3**: This article presents a novel feasibility study protocol focusing on the EPO-Feeding Program. The study aims to assess the feasibility and acceptability of this program, thereby informing interventions to optimize parents' feeding practices in China. This well-designed study addresses a crucial topic and is poised to make a significant contribution to the literature, making it an ideal fit for PLOS One. My comments are, therefore, of a minor nature.

1. The background briefly mentions cultural differences in parental feeding practices, but some insights into the food environment in kindergarten could also be beneficial. For example, do they provide snacks or lunch for the children? If they do, this can also influence children’s food intake. If so, how will this be addressed in the study?

Thank you for this important comment. Yes, we agreed that the kindergarten food environment plays an important role in child eating behaviours. However, our project specifically focuses on the family-based food environment. Additionally, we are targeting parents with children who have started preschool education. Therefore, the influence of the kindergarten context may not be substantial for the included participants since their children already adapted to accepting the snacks or lunch provided at kindergarten. We will assess whether there are significant differences in child age, corresponding to different kindergarten tiers, between the two groups at baseline. Should such differences exist, we will adjust for this covariate in our analysis model. We’ve provided further clarification and detailed specifications as follows.

“It has been widely acknowledged that eating behaviours exert a substantial influence on the risk of childhood overweight and obesity, which can be elucidated within an ecological framework in which children’s characteristics interact dynamically with their surroundings and consequently impact health outcomes [29, 30]. The family, especially primary caregivers (e.g., parents), may influence children’s eating (e.g., food intake) and weight status through feeding practices in the family-based food environment [31, 32]. Hence, many researchers have studied feeding practices to identify efficient strategies to promote children’s healthy eating and prevent childhood obesity in this specific context [2].” (Lines 54-62)

Inclusion criteria: “Parents with at least one preschool child aged 2 to 6 years enrolled in kindergarten (if more than one preschool child, parents are instructed to prioritize the child whose eating habits, weight status, or nutrition they are most concerned about)” (Line 173, Table 1)

2. The sample for this study is intended to include parents proficient in both spoken and written Chinese. However, it is important to consider the parents who may not meet this criterion. Are we assuming that all parents in mainland China are literate? This clarification will help ensure the study's findings are representative.

Thank you. We aim to recruit parents who can speak and write Chinese for effective participation and comprehension as the participants involved will attend sessions, and group discussions, and complete the measurements etc (Table 1). Yes, almost all parents in mainland China are literate. According to the latest available data from United Nations Educational, Scientific and Cultural Organization (UNESCO), the adult literacy rate in China was 97% in 2020 and is estimated to be 99% in 2024.

“Able to speak and write Chinese to attend sessions, group discussions and follow-up assessments”. (Line 173, Table 1)

3. For clarity, please define usual care when first mentioned in the text in line 215.

Thank you. We’ve added it for clarity as you suggested.

“Parents allocated to the intervention group will obtain both the EPO-Feeding Program and usual care (i.e., printed materials containing dietary recommendations for child health published by the Chinese government/Nutrition Society).” (Lines 239-241)

4. Self-reporting questionnaires can have reporting bias, which has been listed as one of the study's limitations. What measures will be undertaken to reduce such biases?

Thank you. We’ve added some details on it.

“To reduce reporting bias, we will ensure anonymity and confidentiality during questionnaire distribution and collection, use clear and neutral wording, provide clear and concise instructions, and incorporate data validation checks such as indirect questions.” (Lines 538-541)

5. What is included in the homework assignment? Could you elaborate more on this?

Thank you. The details of the homework assignment are shown in Table 2. We have also added some information to the main text.

“For instance, participants will be requested to assess their child's weight status using the Chinese 2022 version of growth standards for children under 7 years old and will be encouraged to regularly record their children’s weight status to ensure accurate perceptions of their child’s weight after completing module 1.” (Lines 252-255)

6. A list of the exploratory variables that will be collected could be helpful.

Thank you. We’ve added a list of the exploratory variables.

“The secondary objective aims to evaluate the potential effects of the EPO-Feeding Program on PFPs, PPCW, parenting sense of competence, preschool children’s eating behaviours and weight status.” (Lines 319-321)

**Reviewer #4**: Summary:

The paper evaluates the Empowering Parents to Optimize Feeding Practices (EPO-Feeding Program), a novel intervention aimed at improving parental feeding practices (PFPs) for preschoolers in Yangzhou, China, to mitigate childhood obesity risks. Utilizing a feasibility randomized controlled trial (RCT), it assesses the program's implementation feasibility and acceptability, with secondary outcomes including changes in PFPs, parental perceptions, children's eating behaviors, and BMI-Z scores.

Strengths: The study introduces a novel intervention targeting the critical issue of optimizing PFPs to prevent childhood obesity, a significant public health concern. Utilizes a mix of quantitative and qualitative analyses, including a feasibility RCT, thematic framework analysis, and detailed process evaluation, the authors providing a robust evaluation framework. In addition, based on the Behaviour Change Wheel and Social Cognitive Theory, the intervention is theoretically grounded, enhancing its potential effectiveness.

Comments:

1. Please add the description of measurements of children's height and weight in “4.2 Secondary objectives/outcomes”.

Thank you. We’ve added the relevant information as follows.

“Children's height will be measured to the nearest 0.1 centimetre and weight to the nearest 0.1 kilogram using standardized anthropometric equipment. Trained healthcare teachers will conduct these measurements in the kindergartens during the data collection period, ensuring that measurements will be taken without shoes and with children wearing light clothing.” (Lines 368-372)

2. Please add information on the baseline assessment in the appendix.

Thank you for your suggestion. We have included the details of the baseline assessment in the main text. Please see below. Although we have obtained permission from the original authors to use the questionnaires in this project, we may not possess the copyright authorisation to include complete details or questionnaires in the appendix. Therefore, we believe that providing the baseline questionnaire in the appendix is not feasible.

“The recruitment started on 8^th^ December 2023… Participants were recruited through posters and take-home letters detailing the study. Information regarding the program and participation instructions was disseminated through popular social networks (i.e., WeChat groups and parent meetings). In particular, the participant information sheets, consent forms, and baseline questionnaires (including demographic characteristics, PFPs, PCW, parenting sense of competence, child eating behaviours, and child weight status) were distributed by kindergarten teachers during parent meetings. The teachers also explained the aim of the study to boost parental engagement and emphasized that participation was voluntary, and non-participation would not lead to any negative consequence. JW conducted eligibility screenings of interested parents via phone or in-person interviews. Written informed consent and baseline questionnaires were collected from eligible parents before starting this program.” (Lines 157-169)

“Demographic and socioeconomic data including children’s age, sex, weight, height; parental role, age, weight, height, education level; family structure, household annual income, and number of children were collected at baseline only.” (Lines 294-296)

“The secondary objective aims to evaluate the potential effects of the EPO-Feeding Program on PFPs, PPCW, parenting sense of competence, preschool children’s eating behaviours and weight status. These data will be collected using self-reported tools from participants at three-time points: baseline (T_0_), immediately after the intervention…” (Lines 319-321)

3. Some of the details are wrong, please check carefully:

P2L20, “Backgrounds” changed to “Background”

Thank you. We’ve changed “Backgrounds” to “Background” (Line 23)

P7L139, Yangzhou is a city under Jiangsu Province, please change “Yangzhou District, Jiangsu Province, China” to “Yangzhou, Jiangsu Province, China”

Thank you. We’ve changed “Yangzhou District, Jiangsu Province, China” to “Yangzhou, Jiangsu Province, China”.

**References**

1.	Hoffmann TC, Glasziou PP, Boutron I, Milne R, Perera R, Moher D, et al. Better reporting of interventions: template for intervention description and replication (TIDieR) checklist and guide. Bmj. 2014;348:g1687. doi: 10.1136/bmj.g1687.

2.	Wang J, Chang YS, Wei X, Cao Y, Winkley K. The effectiveness of interventions on changing caregivers' feeding practices with preschool children: A systematic review and meta-analysis. Obes Rev. 2024:e13688. doi: 10.1111/obr.13688.

3.	Hart LM, Damiano SR, Paxton SJ. Confident body, confident child: A randomized controlled trial evaluation of a parenting resource for promoting healthy body image and eating patterns in 2- to 6-year old children. *Int J Eat Disord*. 2016;49(5):458-472. doi: 10.1002/eat.22494.

4.	Hammersley ML, Okely AD, Batterham MJ, Jones RA. An Internet-Based Childhood Obesity Prevention Program (Time2bHealthy) for Parents of Preschool-Aged Children: Randomized Controlled Trial. *J Med Internet Res*. 2019;21(2):e11964. doi: 10.2196/11964.

5.	Haines J, Rifas-Shiman SL, Gross D, McDonald J, Kleinman K, Gillman MW. Randomized trial of a prevention intervention that embeds weight-related messages within a general parenting program. *Obesity (Silver Spring)*. 2016;24(1):191-199. doi: 10.1002/oby.21314.

6.	Leung C, Tsang S, Heung K. The Effectiveness of Healthy Start Home Visit Program: Cluster Randomized Controlled Trial. *Research on Social Work Practice*. 2014;25(3):322-333. doi: 10.1177/1049731514533390.

7.	Pan XF, Wang L, Pan A. Epidemiology and determinants of obesity in China. Lancet Diabetes Endocrinol. 2021;9(6):373-92. doi: 10.1016/S2213-8587(21)00045-0.

8.	Wang J, Zhu B, Wu R, Chang YS, Cao Y, Zhu D. Bidirectional Associations between Parental Non-Responsive Feeding Practices and Child Eating Behaviors: A Systematic Review and Meta-Analysis of Longitudinal Prospective Studies. *Nutrients*. 2022;14(9). doi: 10.3390/nu14091896.

9.	Wang J, Zhu D, Cheng X, Liuzhou Y, Zhu B, Montgomery S, et al. Maternal perception of child weight and concern about child overweight mediates the relationship between child weight and feeding practices. Public Health Nutr. 2022:1-10. doi: 10.1017/S1368980022000040.

10.	Yuan J, Zhang Y, Lu Z, Xu T, Zhang H, Tan Z, et al. Correlation between Children's eating behaviors and caregivers' feeding behaviors among preschool children in China. Appetite. 2019;138:146-52. doi: 10.1016/j.appet.2019.03.022.

11.	Yuan J, Jiang X, Zhu T, Zhang Y, Wang Y, Yang X, et al. Caregivers' feeding behaviour, children's eating behaviour and weight status among children of preschool age in China. J Hum Nutr Diet. 2021;34(5):807-18. doi:10.1111/jhn.12869.

12.	Yuan J, Zhang Y, Xu T, Zhang H, Lu Z, Yang X, et al. Development and Preliminary Evaluation of Chinese Preschoolers' Caregivers' Feeding Behavior Scale. J Acad Nutr Diet. 2019;119(11):1890-1902. doi: 10.1016/j.jand.2019.03.005.

13.	Liu WH, Mallan KM, Mihrshahi S, Daniels LA. Feeding beliefs and practices of Chinese immigrant mothers. Validation of a modified version of the child feeding questionnaire. Appetite. 2014;80:55-60. doi:10.1016/j.appet.2014.04.030.

14.	Collins ME. Body figure perceptions and preferences among preadolescent children. International Journal of Eating Disorders. 1991;10(2):199-208. doi: 10.1002/1098-108X(199103)10:2<199::AID-EAT2260100209>3.0.CO;2-D.

15.	Mareno N. Parental perception of child weight: a concept analysis. J Adv Nurs. 2014;70(1):34-45. doi: 10.1111/jan.12143.

16.	Pallan MJ, Hiam LC, Duda JL, Adab P. Body image, body dissatisfaction and weight status in South Asian children: a cross-sectional study. BMC Public Health. 2011;11:21. doi: 10.1186/1471-2458-11-21.

17.	Wang GH, Tan TX, Cheah CS. Preschool-Age Chinese Children's Weight Status: WHO Classification, Parent Ratings, Child/Family Characteristics. J Pediatr Nurs. 2017;33:63-9. doi: 10.1016/j.pedn.2016.11.005.

18.	Ngai FW, Wai-Chi Chan S, Holroyd E. Translation and validation of a chinese version of the parenting sense of competence scale in chinese mothers. Nurs Res. 2007;56(5):348-54. doi: 10.1097/01.NNR.0000289499.99542.94.

19.	Jiang X, Yang X, Zhang Y, Wang B, Sun L, Shang L. Development and preliminary validation of Chinese preschoolers' eating behavior questionnaire. PLoS One. 2014;9(2):e88255. doi: 10.1371/journal.pone.0088255.

20.	Doyle L, McCabe C, Keogh B, Brady A, McCann M. An overview of the qualitative descriptive design within nursing research. J Res Nurs. 2020;25(5):443-55. doi:10.1177/1744987119880234.

21.	Tong A, Sainsbury P, Craig J. Consolidated criteria for reporting qualitative research (COREQ): a 32-item checklist for interviews and focus groups. Int J Qual Health Care. 2007;19(6):349-57. doi:10.1093/intqhc/mzm042.

22.	Saunders B, Sim J, Kingstone T, Baker S, Waterfield J, Bartlam B, et al. Saturation in qualitative research: exploring its conceptualization and operationalization. Qual Quant. 2018;52(4):1893-907. doi:10.1007/s11135-017-0574-8.

23.	Enders CK. Applied Missing Data Analysis. 2nd ed. New York, NY: Guilford Press; 2022.

24.	Wilcox RR. Introduction to robust estimation and hypothesis testing. 3rd ed. San Diego: Academic Press; 2011.

25.	Sobko T, Brown GTL, Cheng WHG. Does connectedness to nature improve the eating behaviours of pre-schoolers? Emerging evidence from the Play&Grow randomised controlled trial in Hong Kong. *Appetite*. 2020;154:104781. doi: 10.1016/j.appet.2020.104781.

26.	Gomes AI, Barros L, Pereira AI, Roberto MS. Effectiveness of a parental school-based intervention to improve young children's eating patterns: a pilot study. *Public Health Nutr*. 2018;21(13):2485-2496. doi: 10.1017/S1368980018000952.

27.	Haire-Joshu D, Elliott MB, Caito NM, Hessler K, Nanney MS, Hale N, et al. High 5 for Kids: the impact of a home visiting program on fruit and vegetable intake of parents and their preschool children. Prev Med. 2008;47(1):77-82. doi: 10.1016/j.ypmed.2008.03.016.

28.	Hardin JW, Hilbe JM. Generalized Estimating Equations. 1st ed. Boca Raton: Chapman and Hall/CRC; 2002.

29.	Dev DA, McBride BA, Fiese BH, Jones BL, Cho H. Risk factors for overweight/obesity in preschool children: an ecological approach. *Child Obes*. 2013;9(5):399-408. doi: 10.1089/chi.2012.0150.

30.	Wang Y, Cai L, Wu Y, Wilson RF, Weston C, Fawole O, et al. What childhood obesity prevention programmes work? A systematic review and meta-analysis. Obes Rev. 2015;16(7):547-65. doi: 10.1111/obr.12277.

31.	Faith MS, Scanlon KS, Birch LL, Francis LA, Sherry B. Parent-child feeding strategies and their relationships to child eating and weight status. *Obes Res*. 2004;12(11):1711-1722. doi: 10.1038/oby.2004.212.

32.	Ruzicka EB, Darling KE, Sato AF. Controlling child feeding practices and child weight: A systematic review and meta-analysis. *Obes Rev*. 2021;22(3):e13135. doi: 10.1111/obr.13135.
