# Supplementary material for: Empowering parents to optimize feeding practices with preschool children (EPO-Feeding): A study protocol for a feasibility randomized controlled trial
Source: PLoS One. 2024 Jun 3;19(6):e0304707. doi: 10.1371/journal.pone.0304707 (PMC11146728; doi:10.1371/journal.pone.0304707)
Supplement: Attachment — Submitted filename: Editor Response PONE-D-24005284R1 - Accept.docx [file pone.0304707.s008.docx]

PONE-D-24-05284R1

Study Protocol

Title: Empowering Parents to Optimize Feeding Practices with Preschool Children (EPOFeeding): Feasibility Randomized Controlled Trial Protocol for a Group-based Training Program

**Decision:** Accept

Dear Dr. Wang,

Thank you for submitting the protocol entitled “Empowering parents to optimize feeding practices with preschool children (EPOFeeding): A study protocol for a feasibility randomized controlled trial.” The reviewers and I have read the revised manuscript and feel you have been responsive to all the minor revisions requested. As a result, I am pleased to accept this protocol for publication.

I appreciate your hard work on this revision.

Thanks again for considering Plus ONE for your work. We look forward to the revised protocol.

Sincerely,

Dr. Milagros Nores

Academic Editor

PLUS ONE
